# Clinical outcome post treatment of anemia in pregnancy with intravenous versus oral iron therapy: a systematic review and meta-analysis

**DOI:** 10.1038/s41598-023-50234-w

**Published:** 2024-01-02

**Authors:** Anuj Kumar Pandey, Diksha Gautam, Himanshu Tolani, Sutapa Bandyopadhyay Neogi

**Affiliations:** 1 Department of Health Management, International Institute of Health Management Research (IIHMR), New Delhi, India; 2 Institute for Population and Social Research, Mahidol University, Nakhornpathom, Thailand

**Keywords:** Epidemiology, Diseases, Health care, Medical research

## Abstract

Oral iron therapy is often the most common way of treating anaemia; however intravenous iron is considered effective due to rapid iron replenishment. We have dearth of evidence on clinical outcomes post treatment of anaemia. We have searched studies published in English in PubMed, Cochrane, Scopus, ProQuest, and Google Scholar. Our study analysed the clinical outcomes amongst neonates and mother and the adverse events post treatment and assessed the mean change in maternal haemoglobin concentration in both the groups. Forest plots for the clinical outcomes are presented. From a total of 370 studies, 34 Randomized and quasi experimental studies comparing clinical outcomes post-treatment of anaemia in pregnancy were included for quantitative evidence synthesis. Pooled results of maternal clinical outcomes using random effect model [OR: 0.79 (95% CI 0.66; 0.95); 10 outcomes; 17 studies] showed statistically significant difference among both the groups [*Moderate quality evidence]*; however no significant difference [OR: 0.99 (95% CI 0.86; 1.14); 7 outcomes; 8 studies] have been observed for neonatal complications [*Low quality evidence]*. The study found that pregnant women receiving IV iron were significantly less likely to experience adverse events as compared with those receiving oral iron [OR 0.39;  (95% CI 0.26–0.60)]; 34 studies; 13,909 women; [*Low quality evidence*]. Findings from meta-regression analysis showed that IV iron is more likely to reduce maternal complications by 21% compared to oral iron. Increase in odds of adverse maternal outcomes was observed due to increase in gestational age and publication year but no effect for the type of drug used. IV iron increases Hb more and at a higher pace than oral iron. Intravenous iron is more likely to avert adverse maternal outcomes and adverse reactions. However, there is no conclusive evidence on its effectiveness on individual maternal outcome or neonatal outcome/s. Protocol registered with PROSPERO CRD42022368346).

Anemia is a serious public health concern, affecting all age groups. Iron deficiency accounts for 75% of anaemia cases during pregnancy globally^1^. The situation in the developing countries is more serious as close to 95% of the anaemia is due to iron deficiency amongst pregnant women^2^. WHO defines Iron deficiency anaemia (IDA) as haemoglobin (Hb) less than 11 g/dl during pregnancy^3^. IDA is often classified as “mild”, “moderate”, or “severe”, but the Hb cut-off values for these categories vary across geographic settings. According to World Health Organization (WHO), anaemia is recognized as a public health crisis if prevalence is 5.0% or higher. Prevalence of anaemia, equal or more than 40% in a population is classified as a severe public health crisis^4^. Global nutrition target 2025 of World Health Assembly urges all nations to halve the prevalence of anaemia in women of reproductive age^4^. Even after exhaustive efforts made to reduce the burden, it has remained stagnant. No country has been able to achieve the global targets yet^5^. WHO reported that 37% of pregnant women have anaemia worldwide, of which WHO’s South-East Asia (47.8%) and Africa (45.8%) regions are affected the most^6^.

Iron deficiency is more severe in pregnancy because of the increased demand of iron. Majority of iron transfer to the foetus occurs in the second and third trimester of pregnancy^7^. Anemia in pregnancy increases the risk of adverse maternal and neonatal outcomes^8^. It is estimated that anaemia during pregnancy is responsible for around 115,000 maternal deaths (20% of total maternal deaths)^9,10^, and 591,000 prenatal deaths per year globally, of which majority occurred in LMICs, mainly in South Asia and Africa^10^. Iron deficiency anaemia increases the occurrence of low birth weight and preterm birth, which are the major causes of neonatal and infant mortality in LMICs^11–13^. Also, risk of intrauterine growth restriction, low neonatal iron status, preeclampsia, and post-partum haemorrhage among anaemic mothers is high^14^. Therefore, prompt correction of anaemia in pregnancy is a necessity.

IDA can be prevented and corrected by various forms of iron therapy administered through oral, intramuscular, and intravenous routes as also blood transfusion^15^. Oral iron therapy, considered effective in cases of mild and moderate anaemia, has always been the first choice in the past years due to its easy availability, safety, and low cost. However, there is evidence that oral iron therapy is associated with various side effects including metallic taste and gastrointestinal adverse like nausea, vomiting, diarrhoea and constipation, thus hindering patient compliance^3^.

On the other hand, intravenous iron is considered to be more effective due to rapid iron replenishment, particularly in case of severe anaemia and in those who cannot tolerate oral iron therapy. Its use has increased dramatically in the last five years. Major forms of IV iron that are commonly used are iron sucrose, iron dextran, ferric polymaltose, ferric carboxymaltose, etc. However, these preparations are also associated with allergic reactions and anaphylactic shock, as well as venous thrombosis and occasionally cardiac arrest and death^16^.

A Cochrane review by Reveiz L reported that there is insufficient evidence on impact of IV and oral iron treatment during pregnancy on maternal and neonatal clinical outcomes^17^. Although several studies indicate that IV preparations may have better outcomes, emerging evidence suggests no major difference in clinical outcomes^18,19^. Previously published meta-analysis^20^ demonstrated that iron sucrose is more effective in improving the haemoglobin concentration; however there is a dearth of evidence on clinical measures.

Given these inconsistent and insufficient knowledge gaps, we undertook a systematic review and meta-analysis to assess the clinical effectiveness and safety of intravenous iron (intervention) versus standard oral iron (control) in the treatment of women with iron deficiency anaemia during pregnancy.

## Methodology

The study was undertaken in accordance with the Preferred Reporting Items for Systematic Reviews and Meta- Analyses (PRISMA) guideline^21^ ***(Supplementary file 5)*** and the Cochrane Handbook of systematic reviews^22^. The protocol for the review was registered in PROSPERO (CRD42022368346)^23^.

### Search strategy and eligibility criteria

Authors have included five major databases namely: “PubMed, Cochrane, Scopus, ProQuest, and google scholar” for searching the keywords. To ensure that all the literatures are included we have undertaken a detailed examination of cross-references from the identified articles and systematic reviews. The search includes literature published in English until July 2023. A list of reproducible keywords (combination of keywords like ‘iron-deficiency anaemia’, ‘intravenous iron’, ‘adverse events’ and ‘oral iron therapies in pregnant women’) for each database was developed before search *(Supplementary file-1)*. Further for duplication removal, filtering and managing the studies for review/selection we have used Microsoft Excel.

The review included randomized controlled trials (RCT) and quasi-experimental study designs comparing clinical outcomes post-treatment of anaemia in pregnancy with Intravenous versus Oral Iron therapy. Studies were included only if the study participants were assigned to either oral or intravenous arm with any iron formulation. Cohort, case–control, cross-sectional study designs, cross-over studies, case reports, case series, reviews, narrative reviews, systematic reviews, guidelines, and conference proceedings were excluded. Studies fulfilling the eligibility criteria but not reporting the outcome of interest were also be excluded from the study. Also, studies not specific to iron-deficiency anaemia and studies including prophylactic iron administration were also excluded.

### Study selection

The search criteria for the study selection were mutually decided by both the reviewers (AKP and DG) in consultation with the third member (SBN). Post duplication removal, screening of titles and abstracts were done by two primary reviewers (AKP and DG) independently. Any disagreement to finalize the study was resolved by mutual discussion between the reviewers. During deadlock, both reviewers documented the reasons for disagreement and have sought advice from third member (arbitrator-SBN). Full text of the selected articles was retrieved, and both the reviewers have independently reviewed to ascertain the suitability prior to data extraction based on inclusion and exclusion criteria decided a priori. In case full text article was not available, we have written to the authors for sharing the full text. Three consecutive reminders were sent before excluding the article from review. Each exclusion is backed by a reason for removal from the review.

### Data extraction

Post eligibility screening, both the reviewers (AKP and DG) have extracted the data using a pre-defined spreadsheet having the list of essential data elements like study characteristics, patient characteristics (baseline data), and outcome of the treatment or treatment effects *(Supplementary file 2)*. One of the outcomes of the study is mean change in maternal haemoglobin concentration from baseline to defined time period (as mentioned in the selected studies). Other outcomes included clinical outcomes like, adverse maternal and neonatal outcomes and adverse events/side effects amongst study participants. The review followed a standard definition as -

- **Clinical outcomes**: A primary endpoint of potentially life-threatening conditions amongst women and newborn fulfils the criteria for considering the condition as a clinical outcome^24^. The extracted data included maternal outcomes like proportion of women receiving blood transfusion, postpartum haemorrhage, eclampsia, pre-eclampsia, assisted delivery, amount of blood loss, premature labour, hospitalization time, fetal distress syndrome, antepartum haemorrhage, gestational diabetes mellitus and maternal deaths. Neonatal outcomes included gestational age, birthweight, cord pH, APGAR scores, neonatal resuscitation, and neonatal intensive care unit NICU admission.
- **Adverse events/reactions:** Any condition arising as a side effect of the administered drug like nausea, vomiting, metallic taste, rashes etc. Adverse reactions like gastrointestinal complaints, musculoskeletal complaints etc. were also abstracted.

We have abstracted the data in three formats as

- Outcomes have been reported as composite of potentially life threatening maternal/neonatal conditions. This composite outcome implies the presence of any clinical outcome and thus reported as the “total number of women/neonates with one or more clinical outcomes out of total recruited”.
- Other way of reporting the outcome has been for individual components of the composite maternal/neonatal outcomes. For undertaking component specific analysis, we have abstracted outcome specific data. The other format of reporting is the “total number of women experiencing each clinical outcome to all the included women in study”.
- Reporting of the adverse events also follows the similar pattern.

### Risk of bias assessment

Two independent reviewer (AKP and DG) have assessed the quality of the selected article using the Cochrane Collaboration’s tool for assessing the risk of bias in randomised controlled trials^25^. Studies were categorized under three major heads as ‘Low risk’, ‘Unclear’, and ‘High risk’ of bias. Major deadlocks between the reviewers were resolved by discussion with the third member (SBN). We have plotted traffic light plots with {robvis} via rob_traffic_light function in R-studio for depiction of domain specific bias.

### Data analysis

The study assesses the change in haemoglobin (Hb) concentration from baseline to 6^th^ week post-delivery. Studies have reported the mean Hb concentration with standard deviation at baseline, 7^th^ day, 14^th^ day, 21^st^ day, 28^th^ day, at the time of delivery and post-delivery. The days for Hb reporting varied across studies. We have estimated the change in mean of means of the studies from baseline to consecutive days over the time period. We have calculated the delta for both intervention and control arm i.e., mean of mean difference (MD) in Hb concentration from baseline to 7th day, then MD in Hb concentration from baseline to 14th day, 21st day, 28th day, delivery and 6th week post-delivery. In case the mean baseline Hb was > 10gm/dl we have excluded the study from further analysis. Delta is plotted as a trend line for both intervention and control arms to compare the change in Hb concentration over the period.

All the analysis for the study was undertaken in R-studio^26^ using various work packages namely {meta}, {metafor}, {metasens}. For the initial analysis the data of all the included studies were divided as per the type of outcomes amongst the study participants. Results were pooled for meta-analysis separately for each outcome only if ≥ 3 studies reported the same outcome and the overall sample was more than 1000^27,28^. We have calculated the pooled weighted mean differences (WMD) and pooled odds ratio with 95% confidence intervals (CI) using the data from selected studies. We have plotted forest plot to present the pooled estimates for the study outcomes only if the studies were found in sufficient numbers i.e., ≥ 3 studies. We have pooled the data using DerSimonian-Laird random effect models to produce more conservative estimates of the effect size regardless of the evidence of statistical heterogeneity^29^. Between studies heterogeneity was assessed using the Cochran’s Q and Higgins I2 tests^22^. Heterogeneity was considered significant based on conservative thresholds of Q > df for the Q tests or I2 > 30%.

#### Adjusting for the effects of multiple outcomes

For any event reported we assume that every woman has equal probability of experiencing them. For studies reporting the total number of women experiencing multiple outcomes, we have considered the number of outcomes times the women as the denominator. For instance, for a study, with total sample size of ß1 and χ1 outcomes, the total women-outcomes would be χ1 times ß1. Therefore, if the study has reported three types of outcomes namely φ1 = 1, φ2 = 1, and φ3 = 4, we have considered that the entire study sample of ß1 had an equal chance of having the outcome φ1, φ2 and φ3 thus for the study, the total women-outcomes is reported as = ${\text{Total women-outcomes}} = {{(1 + 1 + 4)}} / {(\chi 1 * { \beta }1)}$

### Meta-regression and bubble plot analysis

A regression model, in its most basic form, seeks to predict the value of a second variable y using the value of a first variable. Here in our study meta-regression comprises of individual studies as independent variables (xi’s) and dependent variable (y) represents the effect size of individual studies. Since we can infer that studies with a smaller sampling error have estimates that are closer to the "truth," we also need to make sure that the model in meta-regression gives more weight to those studies. This is accomplished using meta-regression by assuming a mixed-effects model. This model takes into consideration the sampling error and between-study heterogeneity that cause observed studies to diverge from the true overall effect. But more significantly, it also makes use of one or more variables. In our analysis we took five indicators (endline Hb concentration amongst women given IV iron and Oral iron, years of the studies, gestational age as independent variables and conducted meta-regression individually for all indicators affecting effect size i.e., odds of having adverse maternal outcomes in pregnancy.

We used the “bubble” function to display a meta-regression under “meta" package and obtain estimates for effect of each study using “metareg” function. This generates a bubble plot that displays the estimated regression slope and the amount of each study's effect. The bubbles are of different sizes to signify the weight of a study; a larger size denotes a heavier investigation.

### Publication bias and sensitivity analysis

We conducted two sensitivity analysis: The first analysis was done by excluding the study with more than 10% weight in pooled data, since extreme weight might influence the result of the pooled estimate^30^. In the second analysis, we included only those studies with a low risk of bias. Publication bias was tested by contour-enhanced visual funnel plot and selection models like trim-and-fill. In case the number of studies was > 10 we performed egger’s test wherein p value > 0.05 implied no publication bias.

We have assessed the certainty of evidence using the GRADE approach using GRADEpro application^31^. We have categorized the certainty of evidence as ‘high’, ‘moderate’, or ‘low’. The evidence was lowered where the heterogeneity was found to be more than 60%.

## Results

**Figure 1:**
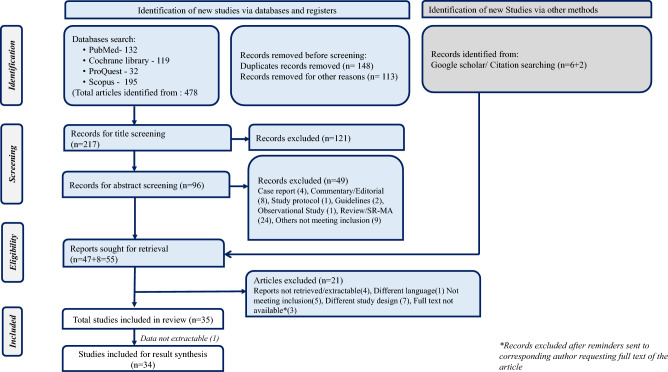
PRISMA flow diagram of studies.

We identified a total of 478 manuscripts. Post duplicate removal, we have identified 217 manuscripts for the title and abstract screening (PRISMA flow diagram Fig. 1). Finally, we have included a total of 34 studies for the review.

The review included 8061 women meeting the inclusion and exclusion criteria. Out of 34 selected studies, 20^24,32–49^ are from Low- and Middle-income countries like India, Pakistan, Bangladesh and Malawi. All the selected 34 studies are randomised controlled trials and were available in full text for review.

Most of them were two-armed RCTs except few. One study performed^50^ RCT using oral iron versus ferric carboxymaltose; and Oral iron versus iron polymaltose. Another one^51^ divided the sample based on the dosage of drug in the iron group (oral iron versus two doses of 200 mg iron sucrose; oral iron versus three doses of 200 mg iron sucrose). Another study^52^ has taken the sample from varied population group (Korean versus Non-Korean study participants). Studies have used various iron formulations and the most commonly used was ferrous sulphate and iron sucrose (n = 27). Other used formulations were ferric carboxymaltose and iron polymaltose.

### Risk of bias assessment

Risk of bias in each study was assessed using the Cochrane Risk of Bias tool^25^. The tool evaluates the studies on five domains namely, randomization and concealment, blinding and deviations from intended interventions, missing outcome data, measurement of the outcome, and selection of the reported result. Amongst the selected 35 studies eligible for the review, 21 studies had low risk of bias, nine studies showed some concern, and five studies have high risk of bias *(Supplementary file 3)*. Post ROB assessment we have removed 1 study^53^ as data was not extractable, leaving a total of 34 studies for review synthesis.

Randomization process was detailed in 22 studies^18,19,54–73^, while insufficient information was available from ten studies^74–83^, no information from two studies^84,85^ while only one study reported the selection through non-random allocation sequence^86^. Allocation concealment was described adequately using opaque envelopes in 12 studies^18,55,60,62–66,68,69,72,76^, while remaining did not indicate allocation concealment in their studies.

Blinding at patient level and provider level was not always feasible owing to different routes of administration. 17 studies did not mention about blinding or open-label in their studies^54,57,59,61,62,64,65,70,72,74–76,78,79,81,82,85^; 13 studies were open-label trials^19,56,58,63,66,67,69,71,73,77,80,83,86^. Two studies reported blinding of data analysts or statisticians^60,68^. One reported blinding of only outcome assessors^18^.

**Figure 2:**
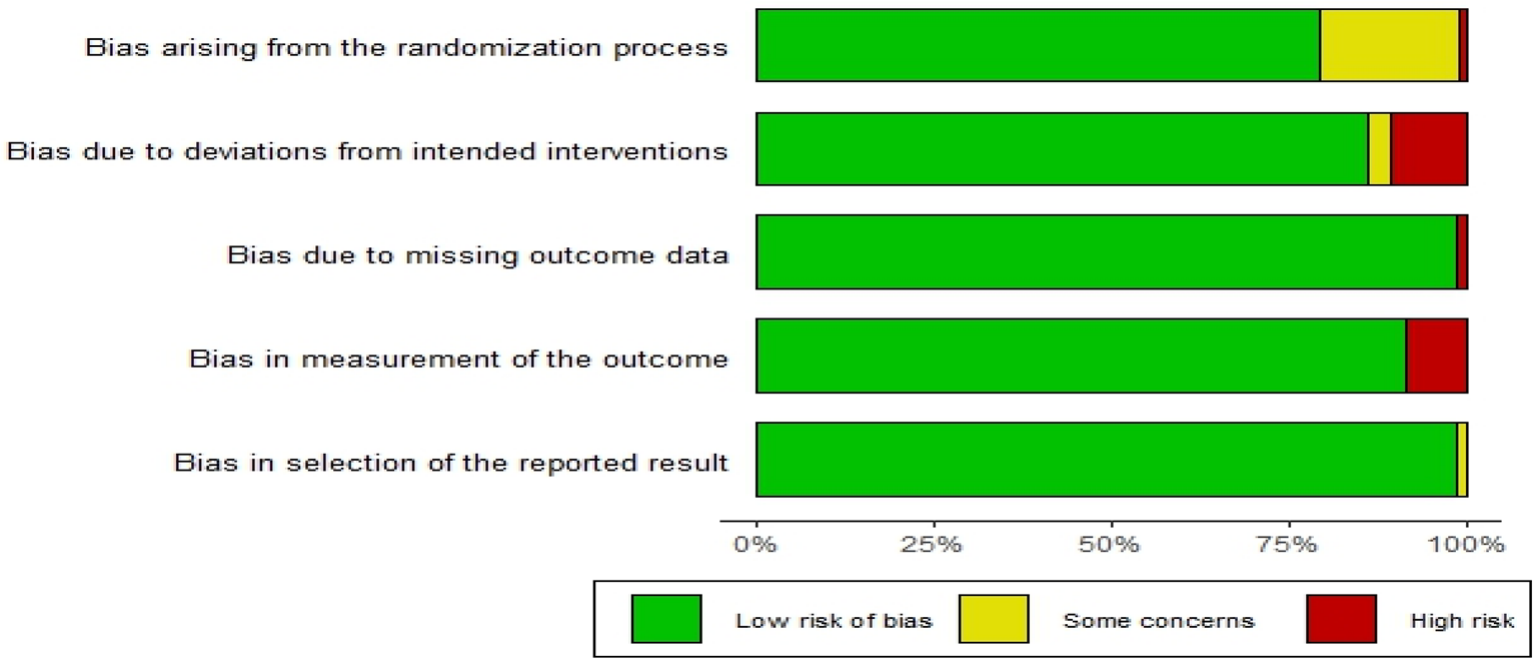
Summary of Risk of Bias: Risk of bias graph about each risk of bias item presented as percentages across all included studies.

Intention-to-treat analyses were used and reported in 9 trials^18,19,55,58,63,70,74,80,83^ to assess outcome measures. Two trials used per-protocol analyses^60,67^, while other studies have not adequately explained the analysis type. All studies reported losses to follow-up and drop-out rates, which were less than 20% in all the studies, except Lewkowitz et al.^60^which reports about 40% loss to follow-up, thus having high risk of bias. Figure 2 summarises the results for risk of bias for each included study.

### Summary of the findings

Forest plots for the clinical outcomes (maternal and neonatal outcomes after treatment with iron preparations) were prepared. Studies with common effect sizes were pooled and were analysed to get the forest plots. Studies have reported a total of 23 and 27 types of neonatal and maternal outcomes respectively. However, we have included 10 maternal and 7 neonatal clinical outcomes for meta-analysis that were most commonly reported and those that have huge clinical significance. We have also performed meta-analysis for adverse events/side-effects on mothers for 29 studies. The clinical outcomes with less than 3 studies with total sample less than 1000 were excluded to get robust pooled effects^27,28,87^.

**Figure 3:**
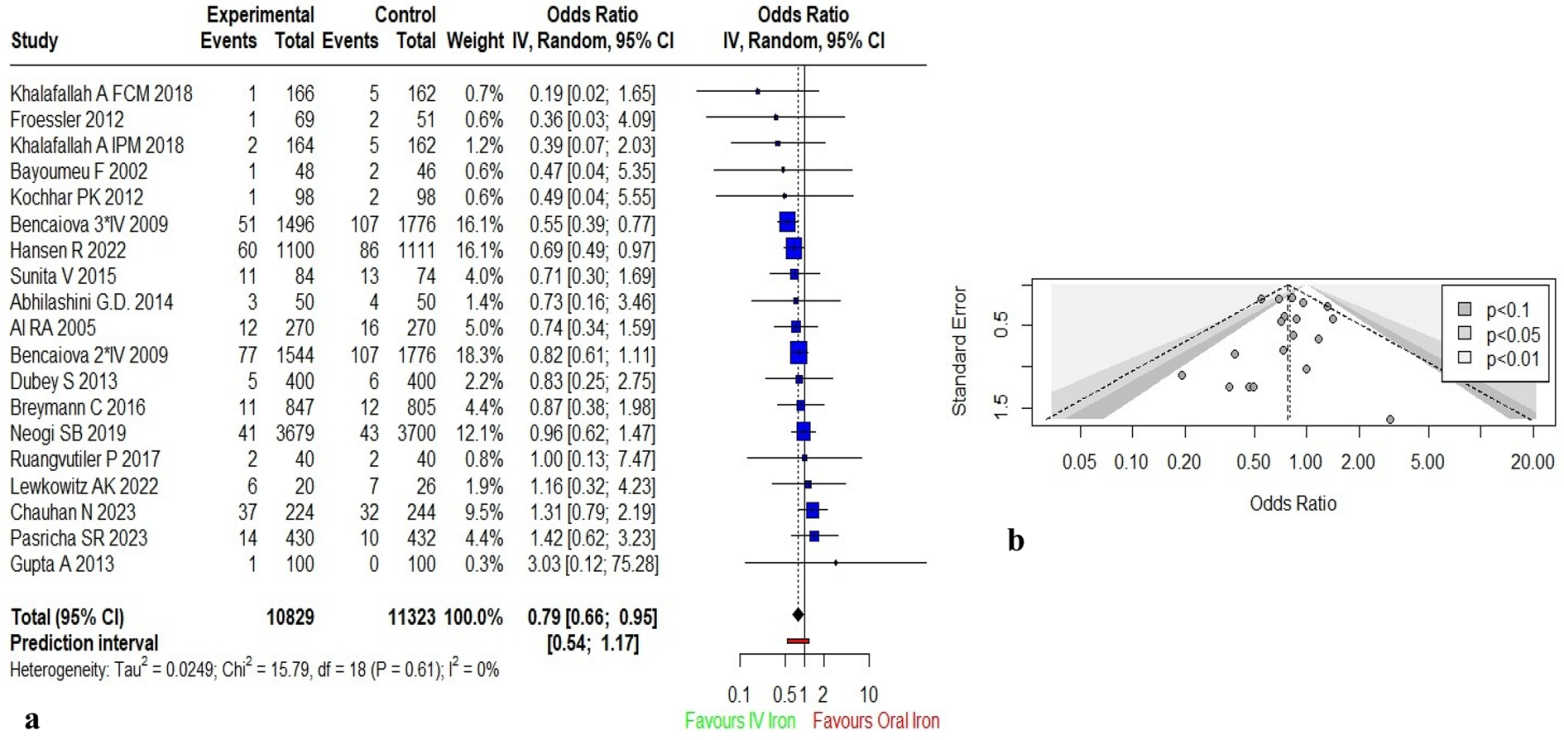
Meta-analysis of effect of IV iron versus oral iron for maternal complications (**a**) Forest plot showing the effect of IV versus oral iron on maternal complications (**b**) Contour enhanced funnel plot for estimates of maternal complications.

Of the total 34 included studies, 17 studies provided data on maternal outcomes(n = 22,152)^24,32,34,39,42,48–51,88–93^. Bencaiova et al.^51^ and Khalafallah et al.^50^ had multiple arms, thus a total of 19 studies were pooled for meta-analysis. Pooled result using random effect model [OR: 0.79 (95% CI: 0.66; 0.95)] showed statistically significant difference among both the groups and illustrated that women receiving IV iron were less likely to experience maternal complications as compared to those who received oral iron *[Moderate quality evidence]*. No significant heterogeneity was observed among the studies (I^2^ = 0%; *p* = 0.61) (Fig. 3a). Sensitivity analysis was performed by excluding studies with high risk of bias at one time and studies with weight > 10% at another time. Removal of study with high risk of bias^60^ revealed no difference in the pooled effect size and heterogeneity. However, removal of studies with weight > 10%^19,63,83^ resulted in change in pooled effect to become non-significant [OR: 0.94 (95% CI: 0.71; 1.24)] which infers that the studies with higher weight were majorly impacting the result.

The contour enhanced funnel plot (Fig. 3b) showed symmetrical distribution, which was confirmed by Egger’s test suggesting no publication bias (*p* = 0.98). 

**Figure 4:**
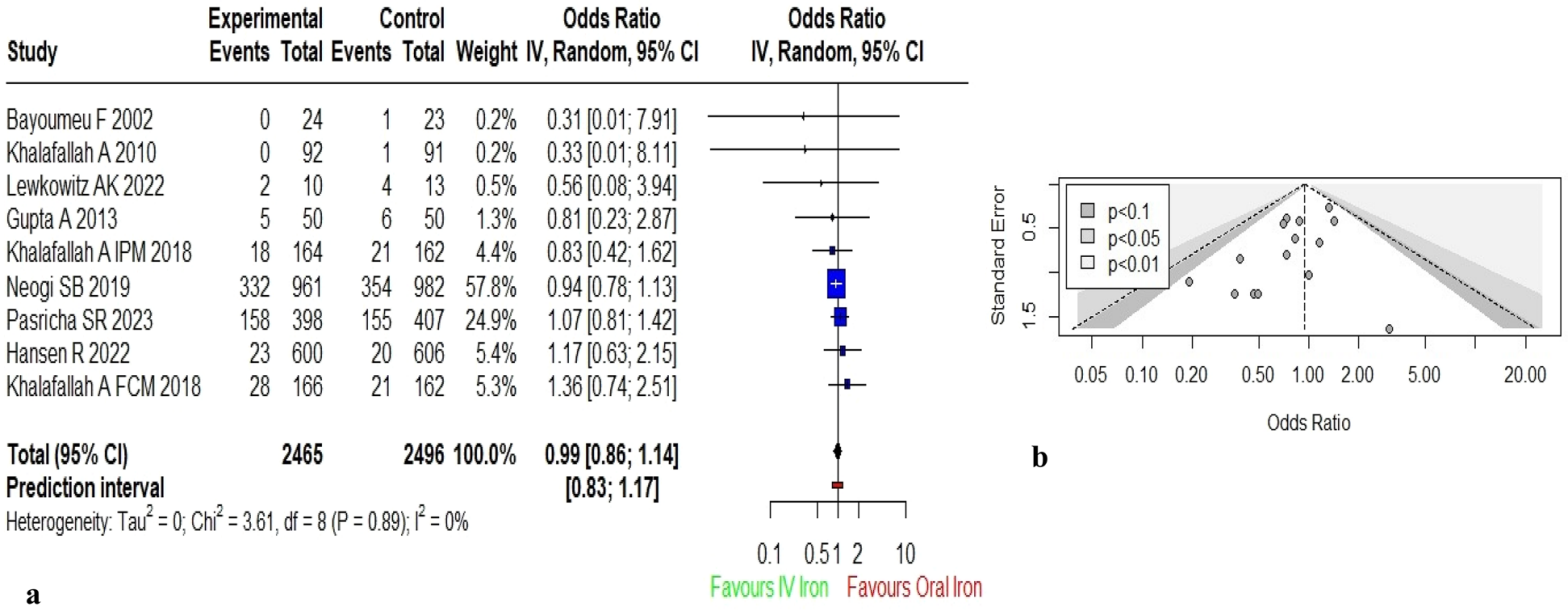
Meta-analysis of effect of IV iron versus oral iron for neonatal complications (**a**) Forest plot showing the effect of IV versus oral iron on neonatal complications (**b**) Contour enhanced funnel plot for estimates of neonatal complications.

Similarly, 8 studies (Fig. 4a) provided the data on neonatal outcomes^24,48–50,88–90,94^ such as need for resuscitation, pre-term birth, low-birth weight, still births, neonatal deaths and macrosomic baby. One two-arm randomised controlled trial that compared two types of IV formulations (FPM and IPM) with oral iron was analysed as two independent studies^50^. Thus, a total of 9 studies (n = 4961) were pooled to conduct the meta-analysis. Utilizing random effect model, pooled result [OR: 0.99 (95% CI: 0.86; 1.14)] showed no significant difference in neonatal complications among IV and oral iron groups *[Low quality evidence]*. No significant heterogeneity was observed among the studies (I^2^ = 0%; p = 0.89). Sensitivity analysis was performed by excluding studies with high risk of bias^60^ at one time and studies with weight > 10%^18,19^ at another time. No change in the pooled effect was observed when high risk study was removed. However, removal of studies with higher weight negligibly altered the pooled effect size [OR: 1.04 (95% CI: 0.74;1.46)].

The contour enhanced funnel plot (Fig. 4b) for neonatal complications showed asymmetrical distribution of studies. Trim and Fill method was applied after filling 4 estimated missing studies, the funnel plot was symmetrical around the adjusted effect size. The adjusted results changed the overall pooled effect to 1.02 [95% CI (0.78; 1.33)].

We have independently assessed the pooled effect of each reported maternal and neonatal outcome if it provided data for three studies or more *(Supplementary file 6)*. Some of the key clinical outcomes are described below.

## Maternal outcomes

### (a) Need for blood transfusion

Out of total 34 studies, blood transfusion was reported in 11 studies (n = 4194)^18,19,55–58,60,63,68,71,83^ Khalafallah A^58^and Bencaiova^63^ presented the findings for both their IV groups i.e., FCM/IPM and 2 doses/3 doses respectively. Thus, a total of 13 studies were included for pooled meta-analysis. Utilizing random effect model, pooled result showed no significant difference between IV group as compared to oral group [OR 0.81 (95% CI [0.53; 1.26) (I^2^ = 0%; *p* = 0.83); *Moderate quality evidence*]. No individual study reported statistically significant effect size.

### (b) Post-partum haemorrhage (PPH)

Seven studies (n = 3655) provided data on PPH^18,19,56,57,63,69,83^. In pooled meta-analysis, utilizing random effects model, the result showed no significant difference in PPH among both the iron formulations (OR 1.01 [95% CI [0.72; 1.41], I^2^ = 0%; *p* = 0.87, *Moderate quality evidence*).

### (c) Caesarean section (CS) and assisted/instrumental delivery

Eight^18,54,55,60,63,71,73,83^and four studies^63,73,80,83^ provided data on the women (n = 1813) who had undergone caesarean section(CS) and assisted/ instrumental delivery respectively. Utilizing random effects model, pooled odds ratio showed no significant difference in the likelihood of CS (OR 1.10; 95% CI [0.86; 1.42], I^2^ = 0%; *p* = 0.93; *Moderate quality evidence*) and assisted/instrumental delivery (OR 1.27 [95% CI [0.70–2.28], I^2^ = 2%; *p* = 0.40; *Moderate quality evidence*) in women receiving IV as compared with those received oral iron.

## (d)Hypertensive disorders

We performed the pooled meta-analysis for the studies that reported any hypertensive disorder and thus, five studies were included. Utilizing random effects model, pooled odds ratio depicted no significant difference between IV iron as compared to oral iron (OR 0.46; 95% CI: [0.20–1.08], I^2^ = 0%; *p* = 0.43; *Moderate quality evidence*).

## Neonatal outcomes

### (a) Birth weight

Eighteen studies reported the mean birth weight of the newborn. Bencaiova et al.^58^, Shim J et al.^52^, Khalafallah A et al.^50^ and Shim JY had reported the findings from both the groups; thus, pooled analysis was performed for a total of 21 studies (n = 3735) reporting birth weight as an outcome. Utilizing random effect model, the pooled results showed no significant difference [SMD: 0.04 (95% CI: 0.03; 0.12), I^2^ = 8%; *p* = 0.36; *High quality evidence*] in the birth weight in both the groups.

### (b) Cord haemoglobin concentration

Utilizing the random effect model, pooled result from meta-analysis from 9 studies (n = 1371) suggests that there is no significant change in the cord haemoglobin [SMD: −0.06 (95% CI: −0.29; 0.17), I^2^ = 71%; *p* < 0.01; *High quality evidence*] concentration amongst women in both the groups.

### (c) Length of newborn

Utilizing the random effect model, pooled findings from 6 studies (n= 1606)  suggests that there is no significant difference in the length of newborn (SMD 0.02, 95% CI -0.01; 0.06, I2 = 0%; p=1.00) [Moderate quality evidence] amongst women in both the groups. Contour enhanced funnel plot showed asymmetrical distribution of studies.

### (d) Still birth and neonatal deaths

Of 34 selected studies, four studies^18,19,70,83^ (n = 3139) provided data on still births/intrauterine death and three studies^18,19,83^ provided data on neonatal deaths. Utilizing random effect model, pooled meta-analysis showed no significant difference in still births [OR: 0.92 (95% CI: 0.55; 1.54); *High quality evidence*]. Similarly, for neonatal deaths (n = 2844) no significant difference was observed (OR 0.72; 95% CI [0.44; 1.18]). Studies showed no significant heterogeneity for both the conditions (I^2^ = 0%; *p* = 0.79 and 0.68; *High quality evidence*).

### (e) Pre-term births

Eight studies^18,19,58,60,64,69,80,83^ provided data on pre-term birth in both the arms. Khalafallah A 2018^58^ reported the outcomes for both of its IV groups (FCM and IPM); thus pooled analysis has been performed for a total of nine studies (n = 3738). Utilizing random effects model, pooled odds ratio showed almost negligible difference among both IV and oral iron arms (OR 0.97; 95% CI [0.79; 1.18], I^2^ = 0%; *p* = 0.84; *Moderate quality evidence*).

###  Adverse events/side-effects in mothers

Of 34 selected studies, 28 studies provided data on adverse events/side effects on mothers in both the groups. Shim JY reported its findings separately for both Korean and non-Korean groups; thus a total of 29 studies (n = 13,909) were considered for pooled meta-analysis. Khalaffallah A 2018^58^ reported side-effects of both the iron formulations but did not compare with oral iron. Similarly, Singh K^74^ mentioned that various side effects were present in women received oral iron but did not reported the values. Thus, these two studies were dropped off from pooled analysis. Seventeen studies reported the number of women who experienced any adverse event and for the remaining 12 studies^32,34,35,38,40,42–44,91,94–96^ we have followed the similar methodology as for the clinical outcomes.

**Figure 5:**
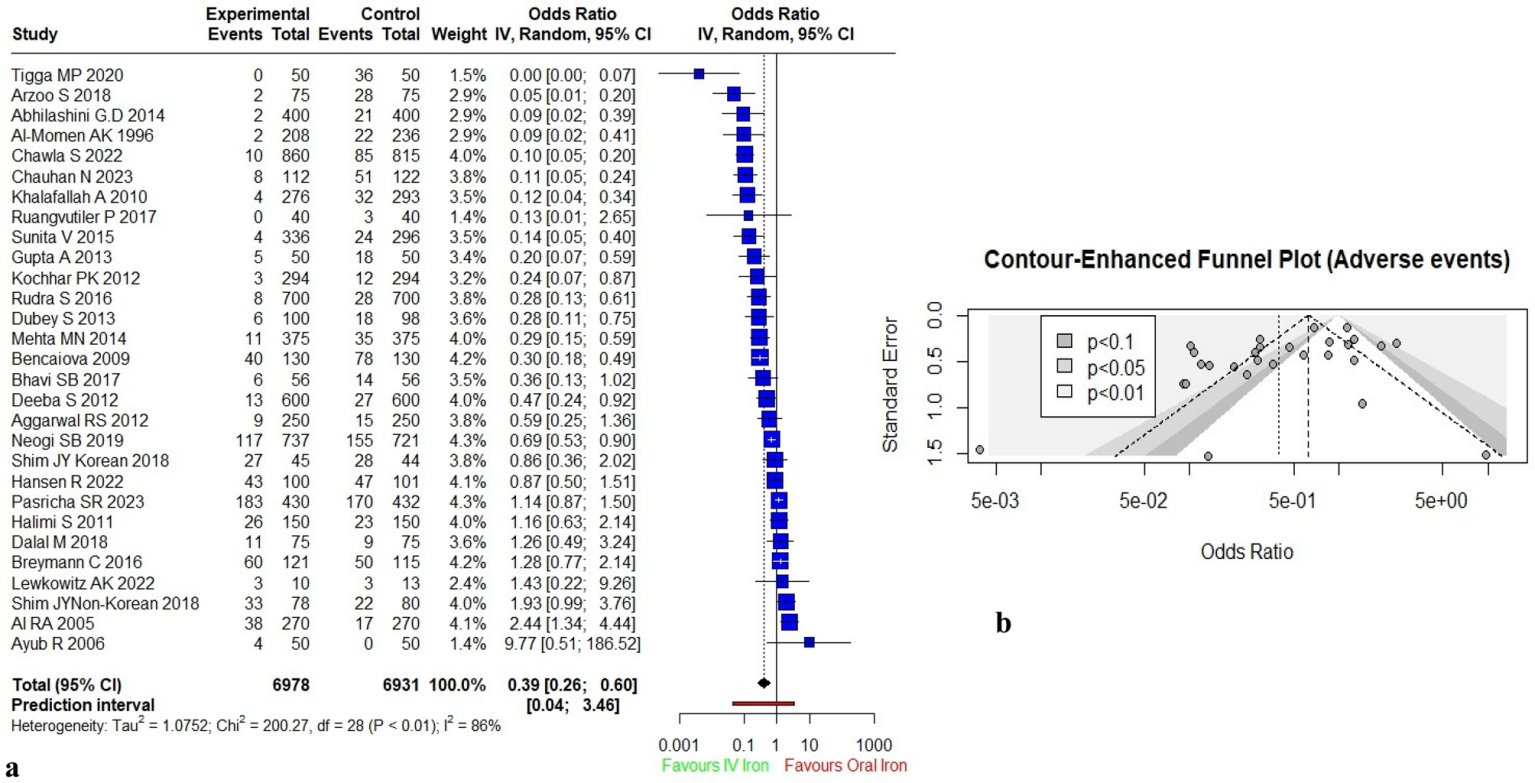
Meta-analysis of effect of IV iron versus oral iron. (**a**) Forest plot showing the effect of IV versus oral iron on adverse events; (**b**) Funnel plot for estimates in meta-analysis for IV iron and adverse events versus oral iron and adverse events.

With random effects model, pregnant women receiving IV iron were significantly less likely to experience adverse events as compared with those receiving oral iron (OR 0.39; 95% CI [0.26–0.60]). Significant heterogeneity was observed (I^2^ = 86%; *p* < 0.01) (Fig. 5a,b). Sensitivity analysis was done by excluding studies with high risk of bias^60,67,84^ that suggested no significant difference in the pooled effect size and heterogeneity. No study was found with weight > 10%. Visual inspection of asymmetry of contour-enhanced funnel plots suggested that publication bias may be present which was confirmed by Egger’s test (*p* < 0.01). We applied the trim and fill method, and after filling 8 estimated missing studies, the funnel plot was found to be symmetrical around the adjusted effect size. The adjusted results changed the overall pooled effect to 0.68 [95% CI (0.41; 1.18)].

###  Rise in  Hb

**Figure 6:**
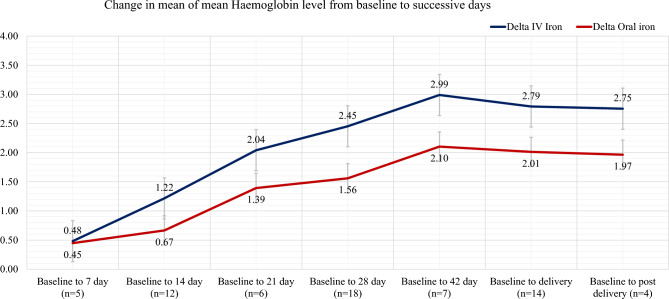
Change in mean of mean Haemoglobin concentration from baseline to successive follow up days. (n indicates the total number of studies reporting the Hb on the respective days).

Mean change in maternal Hb concentration from baseline to 6 week post-delivery was the outcome reported by many studies. We have excluded 5 studies^50,51,88,94,97^ with mean baseline Hb more than 10gm/dl. Number of studies reporting Hb concentration on specific period varied. We found that the mean of mean Hb of the selected studies remained same for both the arm at baseline (mean of x̄Hb_iv_ = 8.54; x̄Hb _or_ = 8.67). However, a difference of mean of mean  Hb represented as delta(δ) (Fig. 6) indicated a rapid rise in Hb levels in IV iron group as compared to oral iron group.

### Meta regression analysis for composite maternal clinical outcomes

Results of the meta regression analysis indicate 10.81% of the difference in true effect sizes of clinical outcome can be explained by the publication year, 4.18% by endline Hb level through oral method and 7.5% by endline Hb level through intravenous method. From the pooled effect size for every additional year, the effect size of a study is expected to rise by 0.008 if *p*-value was significant. We found that increase in minimum gestational age increased the odds of adverse outcomes. Also, no effect was observed for the type of drug used among IV or Oral group on the odds of adverse maternal outcomes.

The estimate of the residual heterogeneity variance which is the variance that is not explained by the predictor endline Hb level for Oral is 0.0051 and that for IV is 0.0107. Although odds ratio of estimated effects for endline Hb level of IV and oral iron are insignificant, yet there is a decline in overall odds ratio of having an adverse outcome (Table 1). More specifically, if the p-value was significant then for one unit rise in endline Hb-level through oral therapy, mean of odds ratio for maternal complications would decrease by 0.21 and for one unit increase in Hb-level through intravenous therapy the mean odds ratio will decrease by 0.13. *Supplementary file 4* provides in-depth explanation of the meta-analysis findings.

**Table 1:** Estimates for Meta-regression.

Regressors	Estimated effect	*p*-value	${\tau }^{2}$^(1)^	${I}^{2}$^(2)^	${H}^{2}$^(3)^	${R}^{2}$^(4)^
Year	0.0088	0.5906	0.0159	10.81%	1.12	0%
Endline HB level for Oral	−0.2171	0.1593	0.0051	4.18%	1.04	32.53%
Endline HB level for Intravenous	−0.1300	0.2718	0.0107	7.5%	1.04	0%
Gestational age	0.0217	0.3470	0.0052	5.23%	1.06	37.21%

^(1)^Estimated amount of residual heterogeneity.

(2^)^ Residual heterogeneity / unaccounted variability.

(3^)^ Unaccounted variability / sampling variability.

^(^4^)^Amount of heterogeneity accounted for.

### Quality assessment

We have assessed the level of certainty of the evidence using the GRADE approach for our study outcomes. The certainty of evidence was found to be high for overall increase in the Hb concentration and moderate for maternal outcomes for studies comparing the women who have received intravenous iron as compared to the oral iron group. The generated evidence also suggests low level of evidence for studies comparing the neonatal outcomes and for having adverse events amongst women who have received intravenous as compared to the oral iron group. We have provided the summary of findings as per the GRADE approach, including the reasons for downgrading the evidence in Table 2. Table 2 only presents the findings of four major outcomes namely overall maternal, neonatal outcomes, adverse events and change in haemoglobin concentration. Individual maternal and neonatal outcomes have also undergone Grading using GRADE approach. (*Supplementary File 7*).

**Table 2:** GRADE summary of findings.

Intravenous iron compared to Oral Iron for Rise in Hb											
Participants (studies) Follow-up			Risk of bias	Inconsistency	Indirectness	Imprecision	Publication bias		Overall certainty of evidence		
8061 (34 RCTs)			Not serious	Not serious	Not serious	Not serious	None		⨁⨁⨁⨁ High		
Intravenous iron compared to Oral Iron for preventing adverse maternal outcome											
Certainty assessment							Summary of findings				
Participants (studies) Follow-up	Risk of bias	Inconsistency	Indirectness	Imprecision	Publication bias	Overall certainty of evidence	Study event rates (%)		Relative effect (95% CI)	Anticipated absolute effects	
							With Oral Iron	With Intravenous iron		Risk with Oral Iron	Risk difference with Intravenous iron
22,152 (19 RCTs)	Not serious	Serious ^a^	Not serious^b^	Not serious	None	⨁⨁⨁◯ Moderate	461/11,323 (4.1%)	337/10,829 (3.1%)	**OR 0.79** (0.66 to 0.95)	41 per 1000	**8 fewer per 1000** (from 13 to 2 fewer)
Intravenous iron compared to Oral Iron for preventing adverse neonatal Outcome											
4961 (9 RCTs)	Not serious	Serious ^a^	Not serious	Serious ^c^	None	⨁⨁◯◯ Low	583/2496 (23.4%)	566/2465 (23.0%)	**OR 0.99** (0.86 to 1.14)	234 per 1000	**2 fewer per 1000** (from 26 fewer to 24 more)
Intravenous iron compared to Oral Iron for preventing adverse events (reactions)											
13,909 (29 RCTs)	Not serious	Serious ^a^	Not serious	Not serious	Publication bias strongly suspected ^c^	⨁⨁◯◯ Low	1071/6931 (15.5%)	678/6978 (9.7%)	**OR 0.39** (0.26 to 0.60)	155 per 1000	**88 fewer per 1000** (from 109 to 56 fewer)

**CI:** confidence interval; **OR:** odds ratio.

**a.** The methodology for reporting the outcomes was not similar. The study sample also varied across the studies.

## Discussion

This systematic review and meta-analysis (34 studies; 8061 women) to assess the effectiveness of IV iron on clinical outcomes during pregnancy shows that IV is more likely to reduce maternal complications by 21% compared to oral iron. However, the same was not observed for neonatal complications. The rise in Hb was significantly greater and faster with IV compared to oral iron at different periods during pregnancy and post-partum period. One unit rise in endline Hb will result in a reduction in odds of maternal complications by 0.21 with oral and 0.13 with IV therapy. Adverse reactions were less likely with IV as compared to oral therapy. An analysis of individual maternal and neonatal complication did not show any significant difference in the two groups.

The systematic review included RCTs adding to homogeneity in terms of study design. The studies were conducted across different geographies in the last 15 years contributing to generalizability. The analysis has looked into the effectiveness on individual component of maternal and neonatal outcome that has never been done earlier. Effects of all adverse outcomes were analysed jointly and individually with separate forest plots indicating relevance of both iron therapies. This could be because none of the studies except one^24^ was conducted with clinical outcomes as the primary outcome and hence not powered to detect such a difference. The primary aim of those studies was to detect a rise in Hb after supplementation.

However, the analysis is fraught with several challenges since the types of outcomes/ complications reported varied across studies. We identified outcomes that were most commonly reported (10 maternaland 7 neonatal). It is likely that we might have missed some important clinical complications. Also, the numbers of complications reported was inconsistent. In order to overcome this problem, we have assumed that every pregnant woman has a risk of developing every outcome analysed in the report. We have therefore calculated women-events for every outcome for ease of computation and further analysis. We have also performed meta-regression to understand the effects of attained endline-Hb level and publication year on the odds-ratio of maternal clinical outcomes. Pooling of individual level data from all studies would have given more reliable estimates but that could not be done due to constraints of resources.

Studies have regarded IV iron a safe option for rapid improvement in anaemia level amongst pregnant women^20,98–103^ which is in line with the result from our meta-analysis. It also highlights faster improvement in the haematological indices after use of intravenous iron.

Several reviews and meta-analyses have been conducted to assess the effectiveness of IV iron in the recent past^20,98–105^. However, all the studies have focussed on the rise in haemoglobin. Several meta-analysis^100,101^ and Cochrane reviews^98,104^ have even concluded that there is a dearth of literature and evidence on clinical outcomes be it maternal or neonatal. Some of the reviews have endeavoured to assess the clinical parameters as a secondary outcome and have reported the overall pooled results^100,104^. These authors have concluded that there is an underreporting of the clinical outcomes amongst women and neonates. Our study findings aims to fill in this gap in the literature by pooling relevant studies.

Unlike other published literature^100,104^, we have included a total of 10 maternal and 7 neonatal clinical outcomes from a total of 34 studies. Our study found that women in the IV iron arm are less likely to experience maternal complications as compared to oral iron, although none of the individual studies demonstrated a significant difference.

Adverse events after iron therapy be it oral or parental iron is an essential phenomenon; we have assessed this parameter and noted that pregnant women receiving IV iron were significantly less likely to experience adverse events as compared with those receiving oral iron. This finding corroborates with other published meta-analysis^20,90,99,103,105^. However another meta-analysis^98^ even suggested having less robust evidence on severe adverse events.

The quality of the study finding is deemed high for overall increase in Hb which is in line with other literature^20^. The quality of the study findings for the primary outcomes namely the maternal clinical outcome was found to be moderate; however the neonatal clinical outcome and overall adverse events amongst the women receiving IV iron have deemed low level of evidence which is in sync with other published literature ^104^.

We also explored the possibilities of identifying the most optimal regimen that could be most cost effective in improving clinical outcomes. Due to the uniformity in the regimens, the same could not be studied. Limited evidence suggests that IV therapy is cost effective for severe anaemia in pregnancy^106^ and also in postpartum anaemia^107^.

To conclude, our analyses address the objective of comparing IV v/s oral iron therapies in pregnancy. It affirms the findings from other studies that IV iron increases Hb more and at a higher pace than oral iron. It is more likely to avert adverse maternal outcomes and adverse reactions. However, there is no conclusive evidence on its effectiveness on individual maternal outcome or neonatal outcome/s. More studies elucidating the effect on critical clinical outcomes are needed to support requisite programmatic and policy level decisions.

### Supplementary Information

Supplementary Information 1.

Supplementary Information 2.

Supplementary Information 3.

Supplementary Information 4.

Supplementary Information 5.

Supplementary Information 6.

Supplementary Information 7.

## Data Availability

All the data and literatures available in public domain were used for the systematic review and meta-analysis. The dataset can be obtained from the corresponding authors on request.
