# Supplementary material for: Clinical outcome post treatment of anemia in pregnancy with intravenous versus oral iron therapy: a systematic review and meta-analysis
Source: Sci Rep. 2024 Jan 2;14:179. doi: 10.1038/s41598-023-50234-w (PMC10761955; doi:10.1038/s41598-023-50234-w)
Supplement: Supplementary file 1 — Supplementary Information 1. [file 41598_2023_50234_MOESM1_ESM.docx]

**Supplementary File 1**

**Table 1. The adjusted search terms as per searched electronic databases**

| **Database** | **No** | **Search Query** | **Results** *(As on 12th Jan’23)* | ***As on 11^th^ July ‘23*** |
| --- | --- | --- | --- | --- |
| ***Cochrane*** | | |  |  |
|  | #1 | (("pregnant women":ti,ab) OR (pregnan*:ti,ab) OR (Antenatal:ti,ab)) | 66,227 | 69,703 |
|  | #2 | (("IV iron sucrose":ti,ab) OR ("intravenous iron therapy":ti,ab) OR ("intravenous iron":ti,ab) OR ("iron sucrose":ti,ab) OR ("ferric carboxymaltose":ti,ab) OR ("iron infusion":ti,ab) OR ("iron sucrose":ti,ab) OR ("iron sucrose injection":ti,ab) OR (IVIS:ti,ab) OR (FCM:ti,ab) OR ("Parenteral iron therapy":ti,ab)) | 1,713 | 1,790 |
|  | #3 | (("Oral iron":ti,ab) OR (Ferrous*:ti,ab) OR (IFA:ti,ab) OR ("iron folic acid":ti,ab) OR ("oral iron therapy":ti,ab)) | 2,856 | 2,992 |
|  | #4 | (("Adverse pregnancy outcome":ti,ab) OR (APO:ti,ab) OR ("maternal outcome":ti,ab) OR (preterm:ti,ab) OR (sepsis:ti,ab) OR ("Postpartum hemorrhage":ti,ab) OR (bleeding:ti,ab) OR (Hemoglobin:ti,ab) OR (Haemoglobin:ti,ab) OR (Anaemia:ti,ab) OR (Anemia:ti,ab) OR ("blood transfusion":ti,ab) OR (Caesarean:ti,ab) OR (c-section:ti,ab) OR ("fetal outcome":ti,ab) OR ("birth outcome":ti,ab) OR ("birth weight":ti,ab) OR (LBW:ti,ab) OR (shock:ti,ab) OR (stillbirth:ti,ab) OR ("still birth":ti,ab) OR ("intrauterine death":ti,ab) OR (IUGR:ti,ab) OR ("intrauterine growth restriction":ti,ab) OR ("neonatal intensive care":ti,ab) OR ("hospital stay":ti,ab) OR ("small for gestation*":ti,ab) OR (jaundice:ti,ab) | 1,61,520 | 1,69,596 |
|  | #5 | #1 AND #2 AND #3 AND #4 | 113 | 119 (6 reviews + 113 trials) |
| ***ProQuest*** | | | |  |
|  | #1 | noft("pregnant women" OR pregnan* OR Antenatal) | 121,119 | 117,450 |
|  | #2 | noft(“IV iron sucrose” OR “intravenous iron therapy” OR “intravenous iron” OR “iron sucrose” OR “ferric carboxymaltose” OR “iron infusion” OR “iron sucrose” OR “iron sucrose injection” OR IVIS OR FCM OR “Parenteral iron therapy”) | 2,989 | 3,019 |
|  | #3 | noft(“Oral iron” OR “Ferrous*” OR IFA OR “iron folic acid” OR “oral iron therapy”) | 7,410 | 7,787 |
|  | #4 | noft(“Adverse pregnancy outcome” OR APO OR “maternal outcome” OR preterm OR Sepsis OR “Postpartum haemorrhage” OR bleeding OR Haemoglobin OR Hemoglobin OR Anaemia OR Anemia OR “Blood transfusion” OR Caesarean OR “C-section” OR “fetal outcome” OR “birth outcome” OR “birth weight” OR LBW OR Shock OR Stillbirth OR “Still birth” OR “intrauterine death” OR IUGR OR “Intrauterine growth restriction” OR “neonatal intensive care” OR “hospital stay” OR “Small for gestation*” OR Jaundice) | 282,843 | 564,456 |
|  | #5 | #1 AND #2 AND #3 AND #4 | 24 | 32 |
| ***PubMed*** | | | |  |
|  | #1 | (("pregnant women"[Title/Abstract]) OR (pregnan*[Title/Abstract])) OR (Antenatal[Title/Abstract]) | 6,14,701 | 6,30,112 |
|  | #2 | (((((((((("IV iron sucrose"[Title/Abstract]) OR ("intravenous iron therapy"[Title/Abstract])) OR ("intravenous iron"[Title/Abstract])) OR ("iron sucrose"[Title/Abstract])) OR ("ferric carboxymaltose"[Title/Abstract])) OR ("iron infusion"[Title/Abstract])) OR ("iron sucrose"[Title/Abstract])) OR ("iron sucrose injection"[Title/Abstract])) OR (IVIS[Title/Abstract])) OR (FCM[Title/Abstract])) OR ("Parenteral iron therapy"[Title/Abstract]) | 12,166 | 12,498 |
|  | #3 | (((("Oral iron") OR (Ferrous*)) OR (IFA)) OR ("iron folic acid")) OR ("oral iron therapy") | 32,906 | 33,702 |
|  | #4 | ((((((((((((((((((((((((((("Adverse pregnancy outcome") OR (APO)) OR ("maternal outcome")) OR (preterm)) OR (sepsis)) OR ("Postpartum hemorrhage")) OR (bleeding)) OR (Hemoglobin)) OR (Haemoglobin)) OR (Anaemia)) OR (Anemia)) OR ("blood transfusion")) OR (Caesarean)) OR (c-section)) OR ("fetal outcome")) OR ("birth outcome")) OR ("birth weight")) OR (LBW)) OR (shock)) OR (stillbirth)) OR ("still birth")) OR ("intrauterine death")) OR (IUGR)) OR ("intrauterine growth restriction")) OR ("neonatal intensive care")) OR ("hospital stay")) OR ("small for gestation*")) OR (jaundice) | 18,60,060 | 19,01,113 |
|  | #5 | #1 AND #2 AND #3 AND #4 | 119 | 132 |
| ***Scopus*** | | | |  |
|  | #1 | TITLE-ABS-KEY ((“pregnant women”) OR (pregnan*) OR (Antenatal)) | 12,73,984 | 12,77,536 |
|  | #3 | TITLE-ABS-KEY ((“IV iron sucrose”) OR (“intravenous iron therapy”) OR (“intravenous iron”) OR (“iron sucrose”) OR (“ferric carboxymaltose”) OR (“iron infusion”) OR (“iron sucrose”) OR (“iron sucrose injection”) OR (IVIS) OR (FCM) OR (“Parenteral iron therapy”)) | 29,385 | 29,471 |
|  | #2 | TITLE-ABS-KEY ((“Oral iron”) OR (Ferrous*) OR (IFA) OR (“Iron folic acid”) OR (“oral iron therapy”)) | 85,632 | 85,743 |
|  | #4 | TITLE-ABS-KEY ((“Adverse pregnancy outcome”) OR (“APO”) OR (“maternal outcome”) OR (“preterm”) OR (“Sepsis”) OR (“Postpartum haemorrhage”) OR (“bleeding”) OR (“Haemoglobin”) OR OR (Hemoglobin) OR (“Anaemia”) OR (“Anemia”) OR (“Blood transfusion”) OR (“Caesarean”) OR (“C-section”) OR (“fetal outcome”) OR (“birth outcome”) OR (“Birth weight”) OR (“LBW”) OR (“Shock”) OR (“Stillbirth”) OR (“Still birth”) OR (“intrauterine death”) OR (“IUGR”) OR (“Intrauterine growth restriction”) OR (“neonatal intensive care”) OR (“Hospital stay”) OR (“Small for gestation*”) OR (“Jaundice”)) | 25,10,166 | 25,22,046 |
|  | #5 | #1 AND #2 AND #3 AND #4 | 195 | 195 |
