# Supplementary material for: Clinical outcome post treatment of anemia in pregnancy with intravenous versus oral iron therapy: a systematic review and meta-analysis
Source: Sci Rep. 2024 Jan 2;14:179. doi: 10.1038/s41598-023-50234-w (PMC10761955; doi:10.1038/s41598-023-50234-w)
Supplement: Supplementary file 2 — Supplementary Information 2. [file 41598_2023_50234_MOESM2_ESM.docx]

**Supplementary file 2:**

**Table 1: Study Characteristics**

| **Author (Year)** | **Country** | **Inclusion criteria** | **IV formulation (SS)** | **Oral formulation (SS)** | **Baseline mean Hb (SD), in g/dl** | **Endline mean Hb mean (SD), in g/dl** |
| --- | --- | --- | --- | --- | --- | --- |
|  |  |  |  |  |  |  |
|  |  |  |  |  |  |  |
| Aggarwal RS (2012) | India | age >18 yrs., GA> 24 wks., anaemia Hb ≤7 gm/dl, transferrin saturation ≤10% and/or serum ferritin ≤15 microg/L. | iron sucrose (25) | iron sulphate (25) | IV: 6.27(0.48)  Oral: 5.95(0.62) | IV: 11.3(0.70)  Oral: 10.26(1.077) |
| Abhilashini G.D (2014) | India | GA- 30-34 wks., Confirmed ID anaemia, Hb 6-8 g/dL | Iron sucrose (N = 50) | Ferrous sulphate (N = 50) | IV: 6.89 (0.6) Oral: 7.16 (0.6) | IV: 10.84 (0.9) Oral: 10.09 (0.7) |
| Al RA (2005) | Turkey | PW 26-34 weeks GA, IDA with Hb 8-10.5 g/dl and ferritin <13 µg/L | iron sucrose (45) | Iron polymaltose complex (45) | IV: 9.9 (0.5)  Oral: 9.8 (0.6) | IV: 12  Oral: 11.3 |
| Al-Momen AK (1996) | Saudi Arabia | PW with GA<32 weeks; IDA with severe iron deficiency anemia with Hb<9g/dl | iron sucrose complex (52) | ferrous sulphate (59) | IV: 7.58 (0.79)  Oral: 7.66 (0.78) | IV: 12.85 (0.66)  Oral: 11.14 (1.24) |
| Bayoumeu F (2002) | France | PW >18 years, Hb 8-10 g/dl, MCV<100 fl at 6 months GA, Ferritin<50 µg/L (<500 mg/600 mg for 3rd trimester PW) | iron sucrose (24) | iron sulphate (23) | IV: 9.6 (0.8)  Oral: 9.7 (0.5) | IV: 11 (1.25)  Oral: 9.7 (0.5) |
| Bhavi SB (2017) | India | PW GA 14-34 wks, Hb 70 to 110 g/L, serum ferritin < 15 ng/ml, age 18 -45 years, singleton pregnancy | iron sucrose (56) | ferrous fumarate (56) | IV: 8.9 (1.07) Oral: 9.14 (1.1) | IV: 10.64 (1.3) Oral: 10.65 (1.03) |
| Khalafallah A FCM (2018) | Australia | PW 2nd & 3rd trimester; Hb ≥8.5 g/dL but ≤12 g/dL; ferritin ≤100 μg/L or TSAT ≤20%; functional iron deficiency (defined by low TSAT <20%); non-anaemic patients, Hb 11-12 g/dL with iron deficiency (as previously defined) | ferric carboxymaltose (83) | ferrous sulphate (81) | IV: 11.33 (0.65) Oral: 11.49 (0.45) | IV: 12.88 Oral: 12.47 |
| Khalafallah A IPM (2018) | Australia | PW 2nd & 3rd trimester; Hb ≥8.5 g/dL but ≤12 g/dL; ferritin ≤100 μg/L or TSAT ≤20%; functional iron deficiency (defined by low TSAT <20%); non-anaemic patients, Hb 11-12 g/dL with iron deficiency (as previously defined) | iron polymaltose (82) | ferrous sulphate (81) | IV: 11.4 (0.54) Oral: 11.49 (0.45) | IV: 12.86 Oral: 12.47 |
| Kochhar PK (2012) | India | PW >18 years, singleton pregnancy, 24-34 weeks GA, moderate IDA (Hb 7-9 g/dl), MCV<85 fL, ferritin<15 ng/mL, no associated complications | iron sucrose (49) | ferrous sulphate (49) | IV: 7.7 (0.5) Oral: 7.6 (0.8) | IV: 13.4 (0.9) Oral: 11.2 (0.9) |
| Lewkowitz AK (2022) | USA | PW with IDA (serum ferritin<30 ng/mL & Hb<10g/dL), singleton pregnancy, 24-34 weeks GA, plan to deliver at one of the study sites. | iron dextran (10) | ferrous sulphate (13) | IV: 9.2 (8.5, 9.6) Oral: 9.6 (9.4, 9.9) | IV: 11 (0.7) Oral: 9.9 (1.1) |
| Mehta MN (2014) | India | PW GA<34 weeks, moderate to severe anaemia (Hb<8 g/dL) | iron sucrose (75) | ferrous sulphate (75) | IV: 6.71 (0.65) Oral: 6.72 (0.67) | IV: 10.64 (0.71) Oral: 10.17 (0.54) |
| Rudra S (2016) | India | PW with GA 24-34 weeks, IDA (Hb 7-9 g/dL) & IDA peripheral smear positive | Iron sucrose (N = 100) | Ferrous ascorbate (N= 100) | IV: 7.81 (0.44) Oral: 7.88 (0.45) | IV: 11.48 (0.61) Oral: 10.90 (0.62) |
| Deeba S (2012) | India | PW 28 - 37 weeks GA with established IDA (Hb 6-10 g%) & serum ferritin < 15 ng/ml. | Iron sucrose (N = 100) | Ferrous ascorbate (N = 100) | IV: 7.9 (0.87) Oral: 7.925 (0.862) | IV: 10.79 (0.84) Oral: 9.903 (0.88) |
| Shim JY (2018) Korean | Korea | Korean PW aged ≥18 years (GA 16–33 weeks), serum ferritin levels ≤20 ng/mL and IDA (Hb 8.0–10.4 g/dL) for GA 16–26 weeks or Hb ≤11.0 g/dL for GA 27–33 weeks)  Non-Korean (Australia, Switzerland, Sweden, Turkey, Singapore, Russia) | ferric carboxymaltose (45) | ferrous sulphate (44) | - | - |
| Shim JY (2018) non-Korean | Non-Korean |  | ferric carboxymaltose (78) | ferrous sulphate (80) | - | - |
| Singh K (1998) | Singapore | PW ≥16 yr., literate, GA 20 - 28 wks., anaemic Hb < 90 gil & IDA with serum ferritin (<20 mg/L), serum iron (< 10 mmol/L), Mean corpuscular Hb< 27 pg, mean corpuscular volume <80 fL. after having excluded thalassaemia. | iron sucrose (50) | iron fumarate (50) | IV: 8.1 (0.1) Oral: 8.6 (0.1) | IV: 11.8 () Oral: 11.2 () |
| Halimi S (2011) | Pakistan | PW with Hb<11 gm/dl, GA 26 to 30 weeks | parenteral iron (50) | ferrous sulphate (50) | IV: 9.20 (1.69) Oral: 9.35 (1.62) | IV: 12.65 (1.06) Oral: 11.20 (0.28) |
| Tigga MP (2020) | India | PW with singleton pregnancy, GA 18 - 28 weeks, IDA- peripheral smear &Hb 7–10.9 g/dL | iron sucrose (50) | ferrous sulfate (50) | IV: 8.84 (0.66) Oral: 9.6 (0.74) | IV: 8.84 (0.66) Oral: 9.6 (0.74) |
| Arzoo S (2018) | Bangladesh | Primigravida or multigravida singleton PW with IDA (Hb < 10gm/dl); 24 - 32 weeks GA | Iron sucrose (N=75) | Ferrous sulfate (N = 75) | IV: 7.96 (0.80) Oral: 7.97 (0.77) | IV: 11.49 (0.39) Oral: 10.39 (0.57) |
| Ayub R (2006) | Pakistan | Iron-deficient PW (GA > 12 weeks), either intolerant or non-compliant with oral iron supplementation. | Iron sucrose (N=50) | Ferrous sulfate (N = 50) | IV: 8.57 (0.9) Oral: 9.5 (0.9) | IV: 11.0 (1.1) Oral: 10.2 (1.2) |
| Bencaiova G (2009) 2*IV | Switzerland | Non-anaemic PW; between 15th and 20th week of gestation; Hb on recruitment was >=10.5 g/dl and they were carrying a single foetus. | Iron sucrose (N=75) | Ferrous sulfate (N = 130) | IV: 12.2 (0.9)  Oral: 12.4 (1.1) | IV: 10.6 (1.8) Oral: 11.0 (1.6) |
| Bencaiova G (2009) 3*IV | Switzerland | non-anaemic PW; between 15th and 20th week of gestation; Hb on recruitment was >=10.5 g/dl and they were carrying a single foetus. | Iron sucrose (N=55) | Ferrous sulfate (N = 130) | IV: 12.2 (0.9)  Oral: 12.4 (1.1) | IV: 11.1 (1.3) Oral: 11.0 (1.6) |
| Ruangvutiler P (2017) | Thailand | Singleton pregnancy; age 18-45 yrs. with IDA anaemia (Hb level <11.0 g/dL or Hct <33.0 %). GA= 33 wks., ferritin level <15 µg/L, CRP of <20 mg/L (15) and whose stool examination revealed no parasites | Iron sucrose (N=40) | Ferrous fumarate (N=40) | IV=9.8 (9.2,10.2) Oral=9.8 (9.3,10.3) | IV= 11.5 (11.0,11.9) Oral=11.6 (10.3,12.2) |
| Breymann C (2016) | Zurich switzerland | PW ≥ 18 years, in 2nd or 3rd T (GW 16–33) and with serum ferritin levels  ≤ 20 ng/mL and IDA [defined as haemoglobin (Hb) 8.0–10.4 g/dL for GW 16–26 or Hb  ≤ 11.0 g/dL for GW 27–33]. | ferric carboxymaltose (FCM) = (N=121) | ferrous sulfate (FS) (N=115) | IV=9.8 (0.8) Oral =9.9 (1.2) |  |
| Chawla S (2022) | India | PW 18-34 wks. GA attending ANC clinic, Hb between 8.5 and <11 gm%. | FCM=(N=172) | FS=(N=163) | IV=9.20±0.44 Oral = 9.17±0.51 | IV=11.9 Oral = 10.77 |
| Dalal M (2018) | India | PW (26 - 34 weeks) of GA with Hb between 7-11 g/dl | iron sucrose=(N=75) | ferrous sulphate (n=75) | IV= 8.40±0.90 Oral=8.42±0.86 | IV=10.16±0.73 Oral=9.05±0.90 |
| Froessler B (2012) | Australia | IDA (Hb<110 g/L and ferritin<12 mg/L [15]) and were hemodynamically stable. PW between GA=28 and 36 Weeks or women within 72 hours of birth either caesarean section or vaginal delivery with blood loss >500 ml. | iron sucrose=(N=100) antenatal cohort (n=69) | Iron sulphate=(n=94) antenatal cohort (n=51) | IV= 10.1 (9.4 - 10.6) Oral= 10.3 (9.3 - 10.7) | IV= 12.7 (11.6 - 13.4) Oral= 12.7 (12.2 - 13.2) |
| Neogi SB ( 2019) | India | Age ≥ 18 yrs., GA- 20 -28 weeks; Hb concentration of 5–8 g/dL, or between 29-32 wks. GA with Hb concentration of 5–9 g/dL, and mean corpuscular volume: RBC count ratio>14 | Intravenous iron sucrose=(N=983) | standard oral iron therapy=(N=1016) | IV= >7g/dl =8·1 (0·6) ≤7 g/dL=6.4 (0·5) Oral=>7g/dl=8·1(0·6) ≤7 g/dL=6·3 (0·6) | - |
| Gupta A (2013) | India | singleton pregnancy (24 and 34 weeks) Hb conc. between 7.0 and 9.0 gm/dL and serum ferritin levels less than 15 ng/mL | iron sucrose (n=50) | ferrous sulphate(n=50) | IV=7.81 ± 0.43 Oral=7.88 ± 0.42 | IV=11.50 ± 0.78 Oral= 10.84 ± 1.12 |
| Neeru S (2012) | India | PW, GA-14 to 36 wks., with Hb level 6.5–10.9 g/dL and ferritin levels less than 27 ng/dL. This cut-off of serum ferritin was chosen because the lower limit in our laboratory is 27 ng/dL | Iron Sucrose group (N=45) | Oral iron group(N=44) | IV= 9.18 (±0.94) (7–10.5) Oral=9.75 (±0.83) (6.8–10.9) | IV= 11.24 (±0.70) (8.9–13.0) Oral=11.06 (±0.63) (9.2–12.2) |
| Khalafallah A (2010) | Australia | Caucasian PW 18 years or above were identified with moderate IDA, defined as Hb <= 115 g ⁄L [reference range (RR) 120–160 g ⁄L] and low iron stores based on a serum ferritin level <30 lg ⁄L (RR 30–440 lg ⁄L). | IV plus oral iron (N=92) | Oral iron (N= 91) | IV plus oral =107.4 (5.4) Oral= 109.3 (4.8) | IV plus oral = 126.6 (9.7) Oral= 121.8 (8.7) |
| Hansen R (2022) | Denmark | women ≥18 years; pw in 2nd trimester (GA 14–21 weeks, inclusive); persistent ID (ferritin<30 µg/L after four weeks of treatment with oral iron) | Ferric derisomaltose (N=100) | Ferrous fumarate (N=101) | IV=12.0±0.9 Oral=11.8±0.9 | IV=12.8 Oral=12.3 |
| Abdelazim I A (2017) | Kuwait | PW >18 years, 24-30 weeks` gestation with hb between 8-10 gm/dl. | Iron Saccharate Complex (N= 126) | Heme Iron Polypeptide (N=124) | IV=8.7 ± 2.5 Oral=8.5 ± 3.5 | IV=11.7 ± 0.9  Oral=11.3 ± 1.3 |
| Pasricha SR (2023) | Malawi | Singleton pregnancy (13 - 26 weeks GA); Hb concentration <10·0 g/dL (moderate or severe anaemia) measured by HemoCue 301+; had no diagnosed inherited red cell disorder and were not clinically judged to require transfusion or have another acute medical illness. | Ferric Carboxymaltose (n= 430 mothers; 395 neonates) | Ferrous sulphate (n= 432 mothers; 401 neonates) | IV=8.83 (1.28) Oral=8.83 (1.23) | IV=11.79 (1.71) Oral=11.61 (1.85) |
| Chauhan N (2023) | India | age 18 - 45 years; singleton pregnancy; gestational age 18-22 weeks; moderate IDA with Hb levels of 7-9g/dl; peripheral smear revealed microcytic-hypochromic anaemia with serum ferritin (<30ng/ml). | Iron Sucrose (n=112) | Ferrous Sulphate (n=122) | IV=8.39 ± 0.5 Oral=8.46 ± 0.37 | 36 wks. gestation: IV=12 ± 1.1 Oral=11.28 ± 0.59 |
| Sunita V (2015) | India | PW >18 yrs.; GA 26 - 32 wks. Hb level between 8 to 10g/dl - sr. Ferritin level <13µg/L | iron sucrose (45) | Ferrous sulphate (45) | IV= 8.7 ± 0.55  Oral= 8.69 ±0.47 | IV= 11.08 ± 0.65  Oral= 10.03 ± 0.46 |
| Dubey S (2013) | India | Haemoglobin level between 7-9g/dl, singleton live pregnancy, gestation age 20-34 weeks, microcytic hypochromic anaemia, serum ferritin level< 15ng/ml. | iron sucrose (100) | Ferrous sulphate (100) | 8.0 ± 0.79 | 8.19 ± 0.66 |

**Table 2: Details of the number of studies in which maternal and neonatal clinical outcomes are reported.**

| **Maternal Clinical Outcomes** | **No. of studies (Study reference)** | **Neonatal Clinical Outcomes** | **No. of studies (Study reference)** |
| --- | --- | --- | --- |
| Blood transfusion | 12 (1–11) | Birth weight (grams) | 18 (1–6,9–20) |
| Post-Partum Haemorrhage (PPH) | 7 (2,4,6,8–10,15) | Gestational age (weeks) | 14 (1,2,4–6,9–13,15–17,19) |
| Caesarean Section delivery (CS) | 8 (1,5,6,9–11,17,21) | Cord blood Hb (g/dl) | 7 (3,9,12,15,16,18,20) |
| Assisted/Instrumental delivery | 4 (6,9,17,20) | Apgar score | 4 (3,4,9,18) |
| Blood loss (ml) | 3 (6,7,9) | Length of baby (cms) | 4 (3,10,18,20) |
| Hospitalization time (days) | 3 (1,6,11) | Neonatal complications | 4 (3,5,8,10) |
| Pre-eclampsia | 4 (1,6,9,20) | Neonatal Hb | 3 (2,4,5) |
| Quality of Life (QoL) | 2 (16,20) | Neonatal resuscitation | 2 (3,9) |
| Vaginal Hematoma | 1 (1) | Preterm birth | 8 (3,5,8–10,13,15,20) |
| Fetal Distress Syndrome | 2 (9,20) | Low-Birth Weight (LBW) | 4 (8–10,13) |
| Pre-term rupture of membrane (PROM) | 1 (20) | Stillbirth/Intra-uterine death | 4 (8–10,16) |
| Threatened labour | 3 (3,6,20) | Neonatal deaths | 3 (8–10) |
| Bronchospasm | 1 (20) | Macrosomic baby | 1 (2) |
| Maternal composite outcome | 2 (8,10) | Head circumference | 1 (3) |
| Puerperal sepsis/infection | 2 (6,8) | Cord pH | 1 (9) |
| Prolonged hospital stays | 1 (8) | Paediatric assistance required | 1 (9) |
| ICU admission | 1 (8) | Neonatal infections | 1 (9) |
| Pruritis gravidarum | 1 (9) | Congenital malformation | 1 (9) |
| Ante-partum hemorrhage (APH) | 1 (9) | Neonatal blood transfusion | 1 (9) |
| Hypertensive disorder of pregnancy | 2 (1,9) | Small for Gestational Age | 1 (10) |
| Gestational hypertension/ Pregnancy Induced Hypertension (PIH) | 3 (6,9,22) | Weight, 4 weeks postpartum | 1 (10) |
| Gestational Diabetes Mellitus (GDM) | 3 (1,6,9) | Venous blood, 4 weeks post-partum | 1 (10) |
| Women achieved target Hb | 1 (17) |  | |
| Thrombocytopenia | 1 (22) |  |  |
| Cholestasis of pregnancy | 1 (22) |  |  |
| Platelet transfusion | 1 (22) |  |  |

**References:**

1. Al RA, Unlubilgin E, Kandemir O, Yalvac S, Cakir L, Haberal A. Intravenous versus oral iron for treatment of anemia in pregnancy: a randomized trial. Obstet Gynecol. 2005 Dec;106(6):1335–40.

2. Bayoumeu F, Subiran-Buisset C, Baka NE, Legagneur H, Monnier-Barbarino P, Laxenaire MC. Iron therapy in iron deficiency anemia in pregnancy: intravenous route versus oral route. Am J Obstet Gynecol. 2002 Mar;186(3):518–22.

3. Khalafallah AA, Hyppa A, Chuang A, Hanna F, Wilson E, Kwok C, et al. A Prospective Randomised Controlled Trial of a Single Intravenous Infusion of Ferric Carboxymaltose vs Single Intravenous Iron Polymaltose or Daily Oral Ferrous Sulphate in the Treatment of Iron Deficiency Anaemia in Pregnancy. Semin Hematol. 2018 Oct;55(4):223–34.

4. Kochhar PK, Kaundal A, Ghosh P. Intravenous iron sucrose versus oral iron in treatment of iron deficiency anemia in pregnancy: A randomized clinical trial. Journal of Obstetrics and Gynaecology Research. 2013;39(2):504–10.

5. Lewkowitz AK, Stout MJ, Cooke E, Deoni SC, D’Sa V, Rouse DJ, et al. Intravenous versus Oral Iron for Iron-Deficiency Anemia in Pregnancy (IVIDA): A Randomized Controlled Trial. Am J Perinatol. 2022 Jun;39(8):808–15.

6. Bencaiova G, von Mandach U, Zimmermann R. Iron prophylaxis in pregnancy: intravenous route versus oral route. Eur J Obstet Gynecol Reprod Biol. 2009 Jun;144(2):135–9.

7. Froessler B, Cocchiaro C, Saadat-Gilani K, Hodyl N, Dekker G. Intravenous iron sucrose versus oral iron ferrous sulfate for antenatal and postpartum iron deficiency anemia: a randomized trial. J Matern Fetal Neonatal Med. 2013 May;26(7):654–9.

8. Neogi SB, Devasenapathy N, Singh R, Bhushan H, Shah D, Divakar H, et al. Safety and effectiveness of intravenous iron sucrose versus standard oral iron therapy in pregnant women with moderate-to-severe anaemia in India: a multicentre, open-label, phase 3, randomised, controlled trial. The Lancet Global Health. 2019 Dec 1;7(12):e1706–16.

9. Hansen R, Sommer VM, Pinborg A, Krebs L, Thomsen LL, Moos T, et al. Intravenous ferric derisomaltose versus oral iron for persistent iron deficient pregnant women: a randomised controlled trial. Arch Gynecol Obstet [Internet]. 2022 Sep 15 [cited 2023 Jul 26]; Available from: https://doi.org/10.1007/s00404-022-06768-x

10. Pasricha SR, Mwangi MN, Moya E, Ataide R, Mzembe G, Harding R, et al. Ferric carboxymaltose versus standard-of-care oral iron to treat second-trimester anaemia in Malawian pregnant women: a randomised controlled trial. The Lancet. 2023 May 13;401(10388):1595–609.

11. Sunita V, Kolekar R, Gundalli S, Nandurkar V. Effectiveness of Intravenous Iron Sucrose versus Oral Iron in Iron Deficiency Anemia in Pregnancy. IOSR Journal of Dental and Medical Sciences (IOSR-JDMS). 2015 Jan;14(1):52–60.

12. Rudra S, Chandna A, Nath J. Comparison of intravenous iron sucrose with oral iron in pregnant women with iron deficiency anaemia. International Journal of Reproduction, Contraception, Obstetrics and Gynecology. 2016;5(3):747–51.

13. Ruangvutilert P, Chanprapaph P, Chuenwattana P, Titapant V, Komoltri C. Low-Dose Weekly Intravenous Iron Sucrose versus Daily Oral Iron for Iron Deficiency Anemia in Late Pregnancy: A Randomized Controlled Trial. JOURNAL OF THE MEDICAL ASSOCIATION OF THAILAND. 2017 May 1;100(5):496.

14. Chawla S, Singh A, Jhamb D, Anupama CH. A Randomised Controlled Trial to Compare Injection Ferric Carboxymaltose and Oral Iron in Treating Iron Deficiency Anemia During Pregnancy. J Obstet Gynaecol India. 2022 Dec;72(6):492–6.

15. Gupta A, Manaktala U, Rathore AM. A Randomised Controlled Trial to Compare Intravenous Iron Sucrose and Oral Iron in Treatment of Iron Deficiency Anemia in Pregnancy. Indian J Hematol Blood Transfus. 2014 Jun;30(2):120–5.

16. Khalafallah A, Dennis A, Bates J, Bates G, Robertson IK, Smith L, et al. A prospective randomized, controlled trial of intravenous versus oral iron for moderate iron deficiency anaemia of pregnancy. J Intern Med. 2010 Sep;268(3):286–95.

17. Chauhan N, Dogra P, Sharma R, Kant S, Soni M. Randomized Controlled Trial Comparing Ferrous Sulfate and Iron Sucrose in Iron Deficiency Anemia in Pregnancy. Cureus. 15(2):e34858.

18. Shim JY, Kim MY, Kim YJ, Lee Y, Lee JJ, Jun JK, et al. Efficacy and safety of ferric carboxymaltose versus ferrous sulfate for iron deficiency anemia during pregnancy: subgroup analysis of Korean women. BMC Pregnancy Childbirth. 2018 Aug 28;18(1):349.

19. Tigga MP, Debbarma AP. A comparative study to evaluate oral iron and intravenous iron sucrose for treatment of anemia in pregnancy in a poor socioeconomic region of Northeast India. Tzu Chi Med J. 2019 Jul 24;32(3):258–61.

20. Breymann C, Milman N, Mezzacasa A, Bernard R, Dudenhausen J, FER-ASAP investigators. Ferric carboxymaltose vs. oral iron in the treatment of pregnant women with iron deficiency anemia: an international, open-label, randomized controlled trial (FER-ASAP). J Perinat Med. 2017 May 24;45(4):443–53.

21. Abhilashini GD, Sagili H, Reddi R. Intravenous Iron Sucrose and Oral Iron for the Treatment of Iron Deficiency Anaemia in Pregnancy. J Clin Diagn Res. 2014 May;8(5):OC04–7.

22. Dubey S, Suri V, Aggarawal N, Das R. Is it safe to use intravenous iron sucrose during pregnancy? A randomized controlled trial. International Journal of Reproduction, Contraception, Obstetrics and Gynecology. 2013;2(4):544–9.
