# Supplementary figures and images for: Clinical outcome post treatment of anemia in pregnancy with intravenous versus oral iron therapy: a systematic review and meta-analysis
Source: Sci Rep. 2024 Jan 2;14:179. doi: 10.1038/s41598-023-50234-w (PMC10761955; doi:10.1038/s41598-023-50234-w)

**Supplementary file 3:**

**Figure 1: Risk of bias assessment for each included study**

*
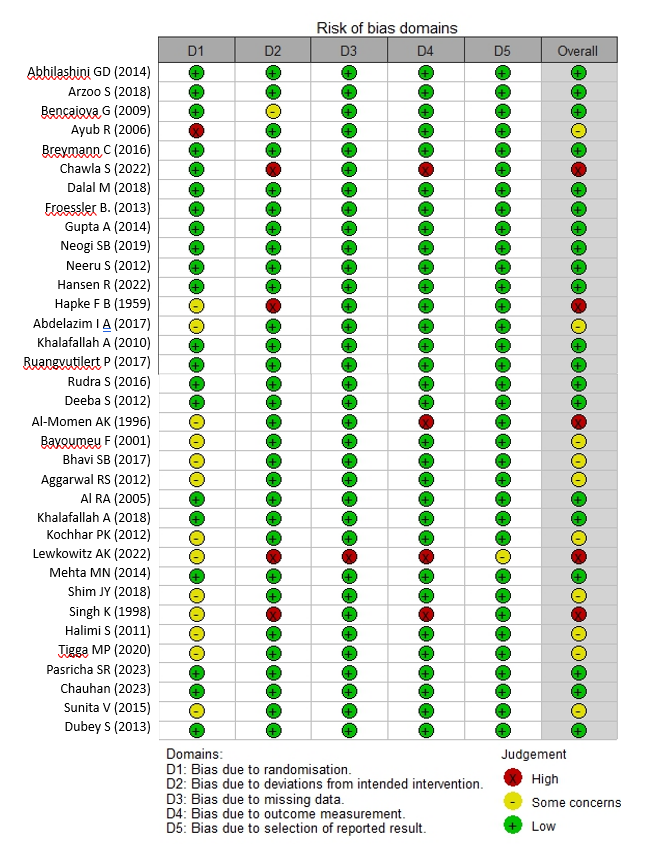
*

Supplement: Supplementary file 3 — Supplementary Information 3. [file 41598_2023_50234_MOESM3_ESM.docx]
