# Supplementary material for: Clinical outcome post treatment of anemia in pregnancy with intravenous versus oral iron therapy: a systematic review and meta-analysis
Source: Sci Rep. 2024 Jan 2;14:179. doi: 10.1038/s41598-023-50234-w (PMC10761955; doi:10.1038/s41598-023-50234-w)
Supplement: Supplementary file 4 — Supplementary Information 4. [file 41598_2023_50234_MOESM4_ESM.docx]

**Supplementary file 4**

**Result of Meta regression analysis:**

**Meta regression analysis for composite maternal clinical outcomes**

Results of the meta regression analysis indicate 10.81% of the difference in true effect sizes of clinical outcome can be explained by the publication year, 4.18% by endline Hb level through oral method and 7.5% by endline HB level through intravenous method. The pooled effect size for every additional year, the effect size of a study is expected to rise by 0.008 if p-value was significant (figure 1).

The estimate of the residual heterogeneity variance, which is the variance that is not explained by the predictor endline Hb level for Oral is 0.0051 and that for intravenous is 0.0107. Although both the estimated effects are insignificant at 5% level of significance but bring down the odds of having an adverse outcome as indicated by bubble plot (Figure 2a, 2b) and negative estimate of the coefficients (Table 2). More specifically, if the p-value was significant then for one unit rise in endline Hb-level through oral therapy, mean of odds ratio for maternal complications would decrease by 0.21 and for one unit increase in Hb-level through intravenous therapy the mean odds ratio will decrease by 0.13.

The reason for insignificance is attributed to the fact that odds of having an adverse outcome may depend on numerous influencers and not only endline Hb levels. Also, because reported Hb-levels don’t have large variation among studies or ranges between 9 to 12 gm/dl, the rise or fall in Hb levels can’t fall outside these limits which might be one of the reasons for the insignificance observed. The aim of the present study was to assess the variation in effect sizes impacted by oral and intravenous therapies, taking endline levels of HB and Minimum Gestational Age (GA) as indicators. Meta-regression analysis for this study creates the need for looking at other factors influencing odds of having adverse outcomes other than effects of HB-level. Minimum Gestational Age (GA) was also considered as one of the independent variables for the meta regression for studies corresponding to which GA interval was available. Variation in the true effect sizes explained by minimum GA is 5.23%. Figure 2c depicts the meta regression ^
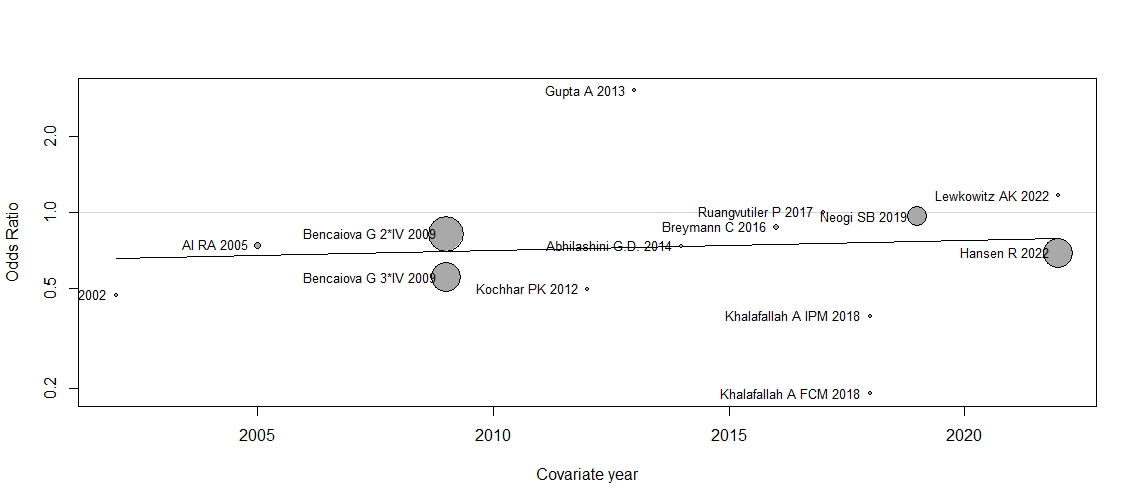
^line due to minimum GA level.

Figure 1: Bubble plot taking different study years as independent variable.


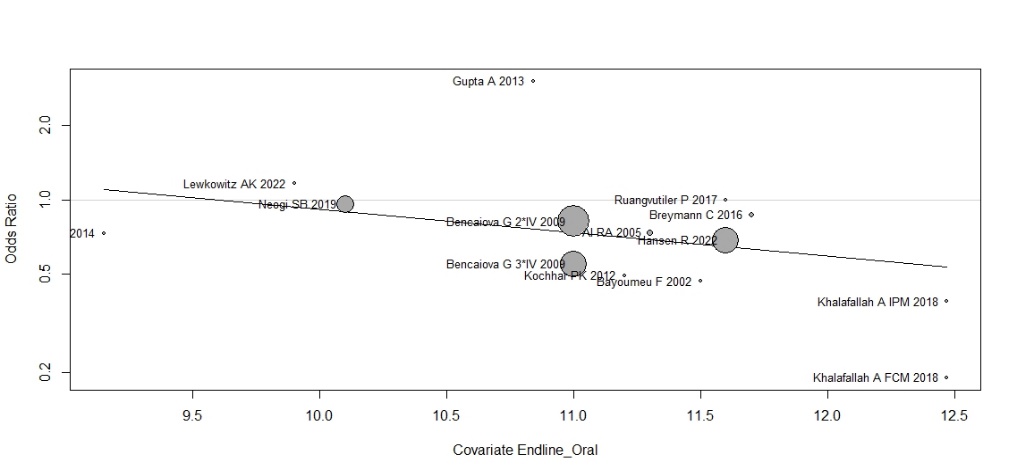


Figure 2a: Bubble plot taking reported Endline HB-level through Oral method as dependent variable.


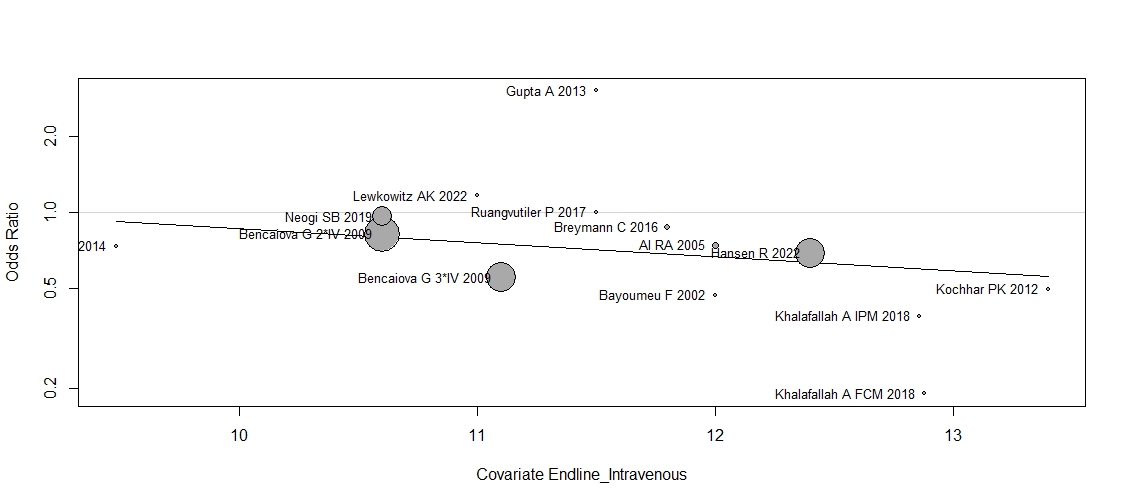
Figure 2b: Bubble plot taking reported Endline HB-level through Intravenous iron method as dependent variable.


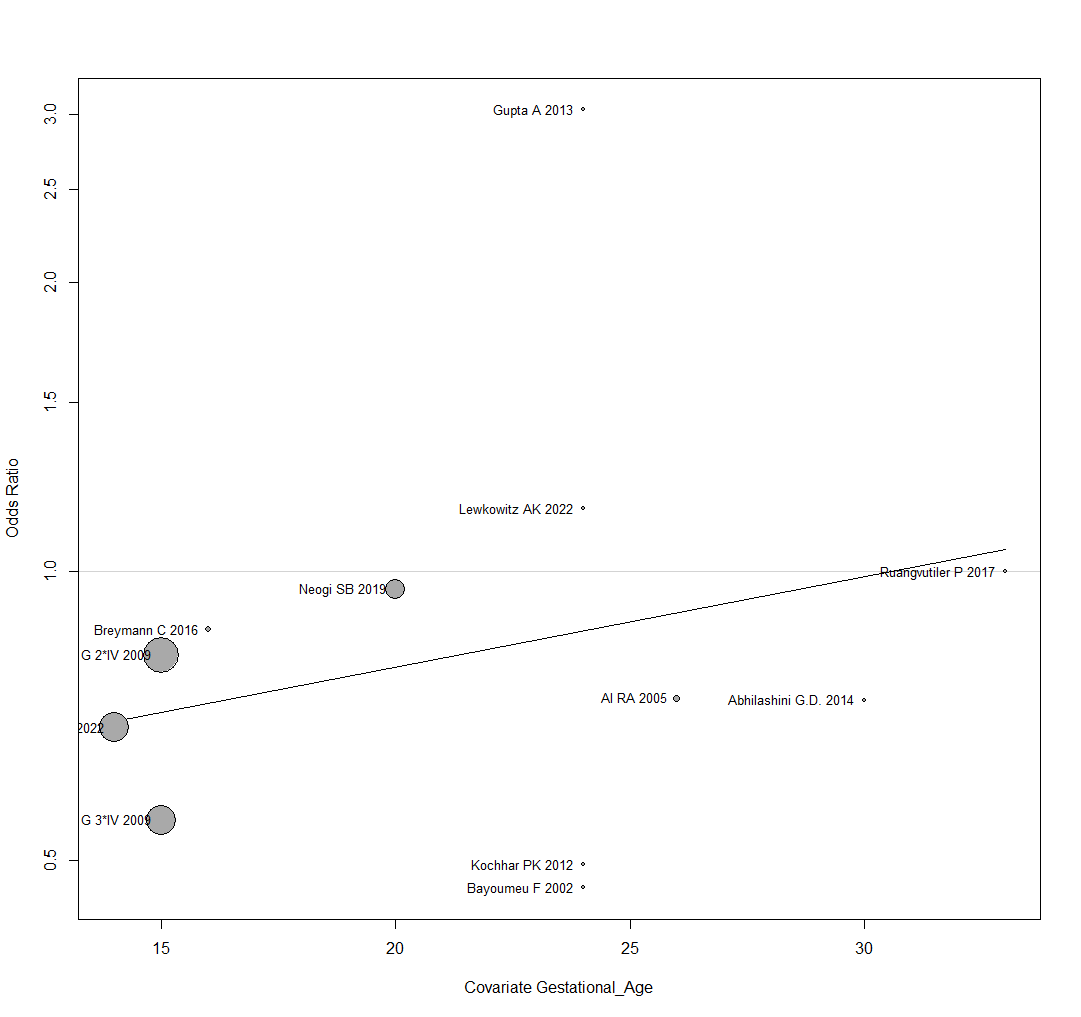


Figure 2c: Bubble plot taking minimum of gestational age as independent variable.
