# Supplementary material for: Clinical outcome post treatment of anemia in pregnancy with intravenous versus oral iron therapy: a systematic review and meta-analysis
Source: Sci Rep. 2024 Jan 2;14:179. doi: 10.1038/s41598-023-50234-w (PMC10761955; doi:10.1038/s41598-023-50234-w)
Supplement: Supplementary file 6 — Supplementary Information 6. [file 41598_2023_50234_MOESM6_ESM.docx]

**Supplementary file 6:** Individual maternal and neonatal complications analysis

**Individual Maternal complications:**

1. **Need for Blood Transfusion:**

Out of total 34 studies, blood transfusion was reported in 11 studies (n= 4194)(1–11) Khalafallah A (4)and Bencaiova (6) presented the findings for both their IV groups i.e., FCM/IPM and 2 doses/3 doses respectively. Thus, a total of 13 studies (Figure 1a) were included for pooled meta-analysis. Utilizing random effect model, pooled result showed no significant difference between IV group as compared to oral group [OR 0.81 (95% CI [0.53; 1.26)]. No individual study reported statistically significant effect size. No significant heterogeneity was observed among the studies (I^2^ = 0%; p = 0.83). Sensitivity analysis with the removal of the study with high risk of bias(5) showed no substantial difference in the pooled effect from the main model. However, removal of studies with higher weight (8,10) highly altered the pooled effect to 0.37 (95% CI: 0.15;0.88) and the observed risk became statistically significant. (Figure 1b)

Studies showed asymmetry in its distribution through contour enhanced funnel plot, with more studies cluttered with less odds ratio and high standard error, thus reflecting the presence of publication bias, which was further confirmed by Egger’s test (p <0.001). Trim and fill effect added 6 missing studies, and it corrected the effect size attenuated to 0.97 (95% CI: 0.65; 1.47), which was not significant (p = 0.91).


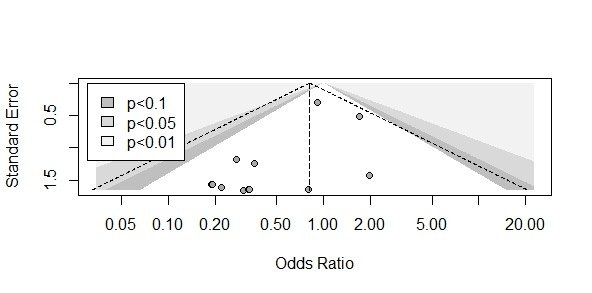

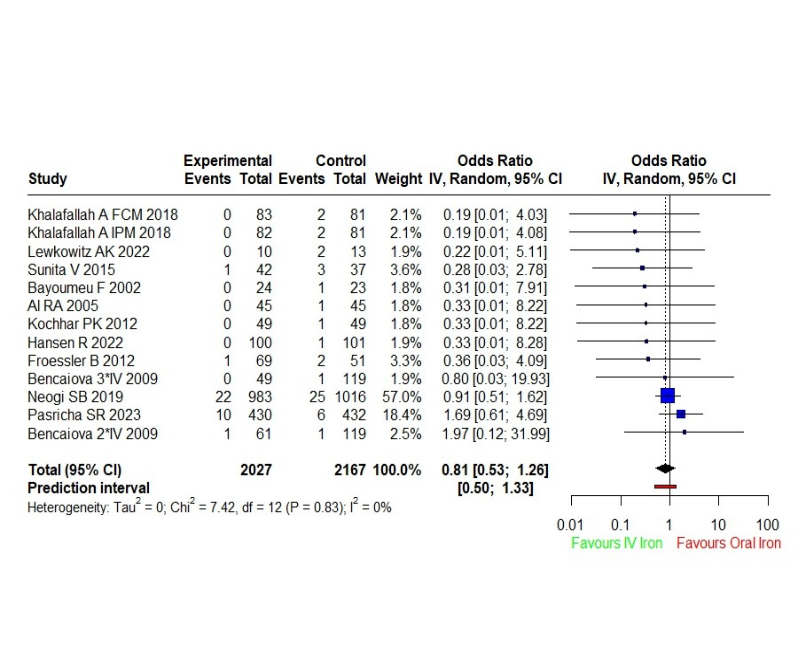


**aa**

**b**

Figure 1. Meta-analysis of effect of IV iron versus oral iron. **a.** Forest plot showing the effect of IV versus oral iron on blood transfusion; **b.** Funnel plot for estimates for blood transfusion.

1. **Post-Partum Haemorrhage (PPH):**

Seven studies (n= 3655) provided data on PPH (2,3,6,8,10–12). Study by Singh K(13) reported in study that there is no significant differences in PPH between the groups but no data were provided on request and hence excluded. Bencaiova (6) presented the findings for both of its groups: 2 dose of IV iron vs oral iron and 3 dose of IV iron vs oral iron, thus a total of eight studies were included in the analysis. In pooled meta-analysis, utilizing random effects model, the result showed no significant difference in PPH among both the iron formulations (OR 1.01 [95% CI [0.72; 1.41]) (Figure 2a). No individual study showed statistically significant effect sizes. No significant heterogeneity was observed among the studies (I^2^ = 0%; p=0.87) (Figure 2b). It is interesting to note that in the study Bencaiova (6) increasing the dose of IV iron from 2 to 3 decreased the odds of PPH among women compared to oral iron, however the effect was found non-significant. Studies showed asymmetry in its distribution through visual funnel plot. We found similar estimates of effect for the sensitivity analysis when performed by removing studies with higher weight.(6,10,11). Studies showed asymmetry in its distribution through contour enhanced visual funnel plot. One study was imputed through trim and fill method which did not show any substantial difference in the pooled effect from the original model [OR 0.99 (95% CI:
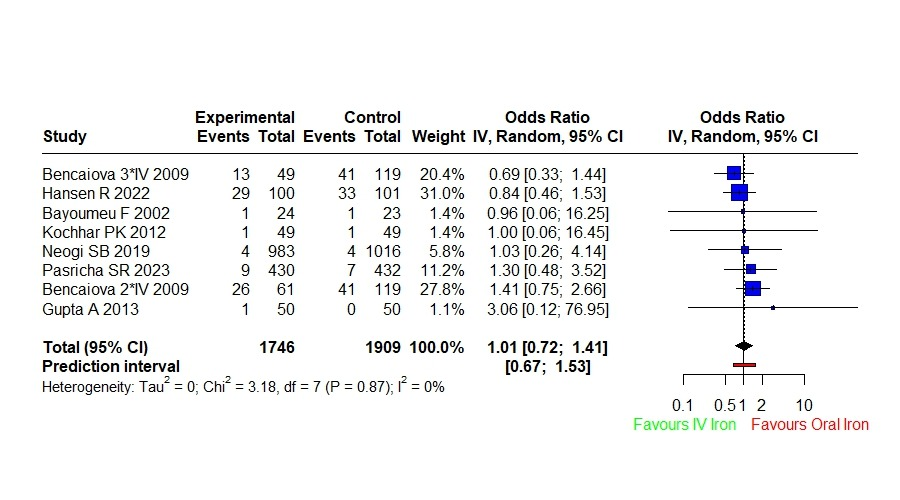

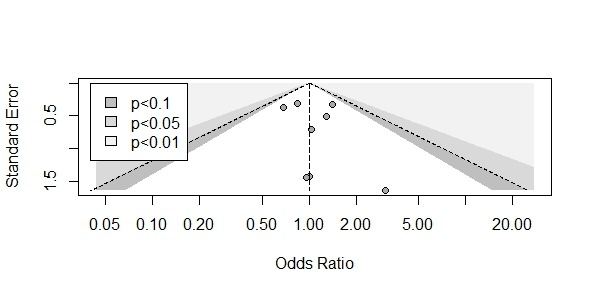
0.71;1.38)].

**aa**

**b**

Figure 2. Meta-analysis of effect of IV iron versus oral iron. a. Forest plot showing the effect of IV versus oral iron on PPH; b. Funnel plot for estimates in meta-analysis for IV iron and PPH versus oral iron and PPH.

1. **Caesarean Section (CS) and Assisted/Instrumental Delivery:**

Eight (1,5,6,9–11,14,15)and four studies (6,11,15,16) provided data on the women (n=1813) undergone caesarean section and assisted/ instrumental delivery respectively. Study by Singh K(13) and Dubey (17) reported no significant differences in mode of delivery but no data were provided on request and therefore we excluded it. Bencaiova (6) reported the outcome for both of its IV groups (2 dose and 3 dose) in both the outcomes, thus pooled analysis has been performed for nine for CS and five for assisted/ instrumental delivery studies.

Utilizing random effects model, pooled odds ratio showed no significant difference in the likelihood of CS (OR 1.10; 95% CI [0.86; 1.42]) and assisted/instrumental delivery (OR 1.27 [95% CI [0.70-2.28])- (Figure 3a and 3c), in women receiving IV as compared with those received oral iron. No individual study showed statistically significant effect sizes for both the outcomes. No significant heterogeneity was observed among the studies for CS (I^2^ = 0%; p=0.93) and assisted/instrumental delivery (I^2^ = 2%; p=0.40) (Figure 3b and 3d). However, the power of the heterogeneity test was very low in assisted/instrumental delivery, as there were only five studies.

Sensitivity analysis for CS deliveries and assisted/instrumental delivery was done by two ways: removing studies with high risk and high weight (>10%). In CS deliveries, removal of studies by both the ways (5,6,10,15) revealed negligible difference in the pooled effect size from the main model, indicating the robustness of the main model. However, removal of studies with weight >10%(6,11,15) in assisted/instrumental deliveries showed a shift of pooled effect size to 0.58 (95% CI: 0.14;2.48), which was non-significant, indicating that studies with higher weight were impacting the overall result.

The contour-enhanced funnel plot showed symmetrical distribution of studies for CS. However, it was asymmetrical distribution of the studies for assisted/ instrumental delivery. Trim and fill effect for assisted/ instrumental delivery imputed estimates from 2 missing studies which shifted the pooled effect size to 1.46 (95% CI: 0.86; 2.48).


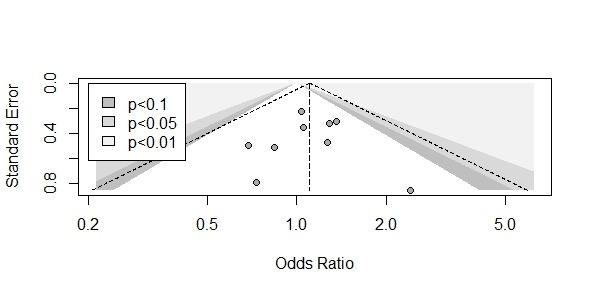

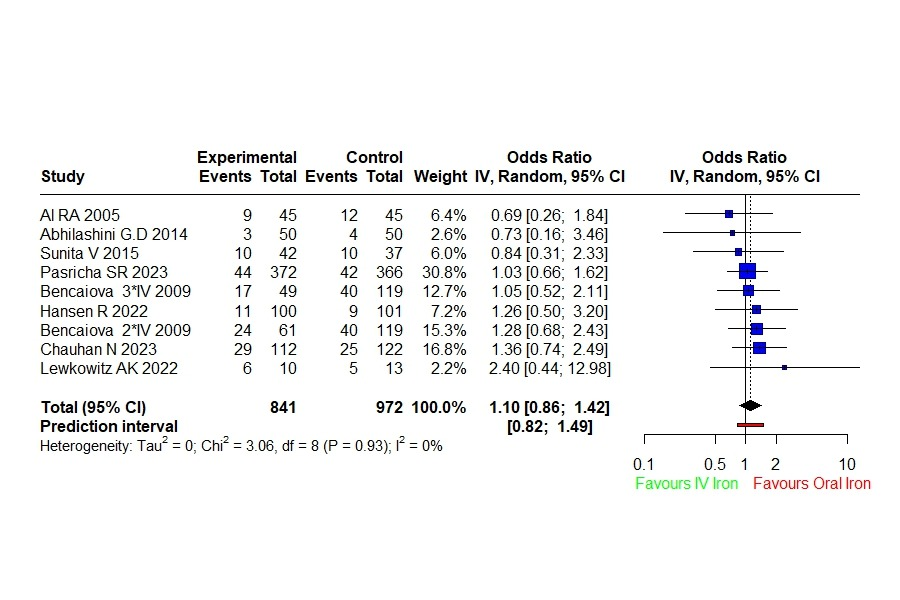


**aa**

**b**


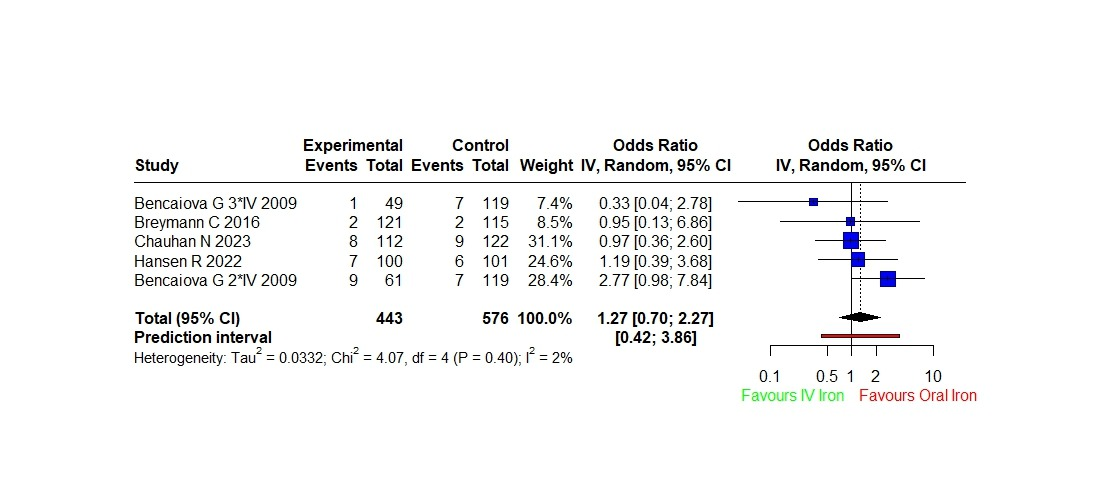


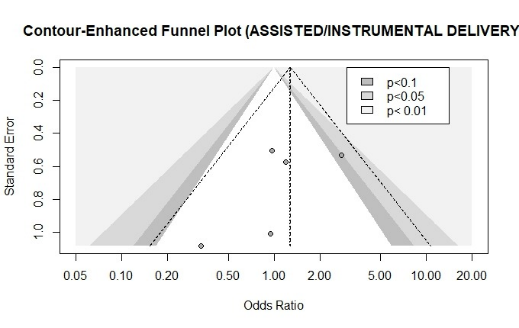


**d**

**c**

Figure 3 Meta-analysis of effect of IV iron versus oral iron. **a.** Forest plot showing the effect of IV versus oral iron on caesarean section; **b.** Funnel plot for estimates in meta-analysis for IV iron and caesarean section versus oral iron and caesarean section**; c.** Forest plot showing the effect of IV versus oral iron on assisted/ instrumental delivery; **d.** Funnel plot for estimates in meta-analysis for IV iron and assisted/ instrumental delivery versus oral iron and assisted/ instrumental delivery.

1. **Hypertensive disorders:**

Five studies (1,6,11,16,17) (n= 987) provided data on some form of hypertensive disorder, out of which overall hypertensive disorders were reported in two studies; pre-eclampsia (1,6,11,16) was reported in four studies; and gestational hypertension or pregnancy-induced hypertension (PIH) (6,11,17) was reported in three studies.

We performed the pooled meta-analysis for the studies that reported any hypertensive disorder and thus, five studies were included. Utilizing random effects model, pooled odds ratio depicted no significant difference between IV iron as compared to oral iron (OR 0.46; 95% CI: [0.20-1.08]) (Figure 4a). We could not observe significant heterogeneity among the studies (I^2^ = 0%; p=0.43) (Figure 4b). However, the power of the heterogeneity test was very low, as there were only five studies. Sensitivity analysis was conducted by removing the studies with weight >10% (6,11,17). After exclusion of the studies, the pooled effect showed that the odds of hypertensive disorders are more in women received IV iron as compared to oral iron, which was opposite the finding from the main model, though the difference among both the arms was non-significant [OR 1.34 (95% CI: 0.09;21.02)] with the presence of non-significant heterogeneity (I^2^ =35%). It is showing that the higher weight studies had a significant impact in the pooled effect.


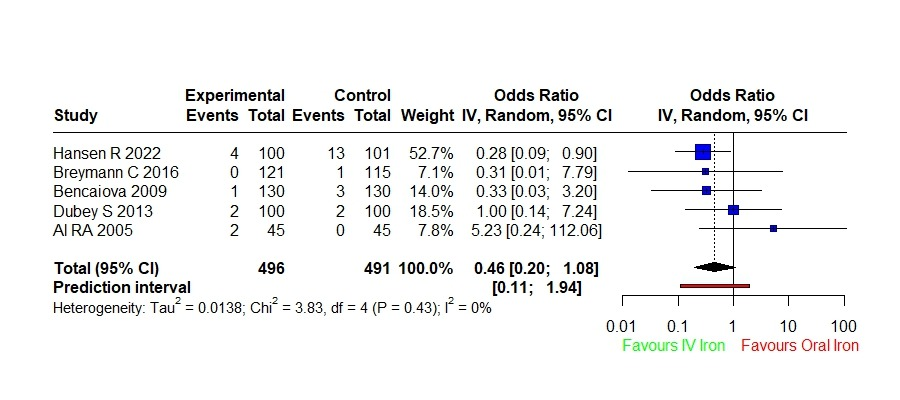
Publication bias is alleged as contour enhanced visual funnel plot shows asymmetrical distribution of studies. Imputation of one study through trim and fill effect decreased the observed effect became nominally significant [OR 0.37 (95% CI: 0.16; 0.84)].


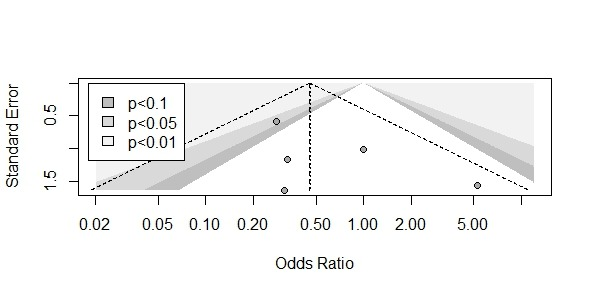


**aa**

**b**

Figure 4 Meta-analysis of effect of IV iron versus oral iron. **a.** *Forest plot showing the effect of IV versus oral iron on hypertensive disorders;* ***b.*** *Funnel plot for estimates in meta-analysis for IV iron and hypertensive disorders versus oral iron and hypertensive disorders.*

**Individual Neonatal outcomes:**

1. **Birth weight:**


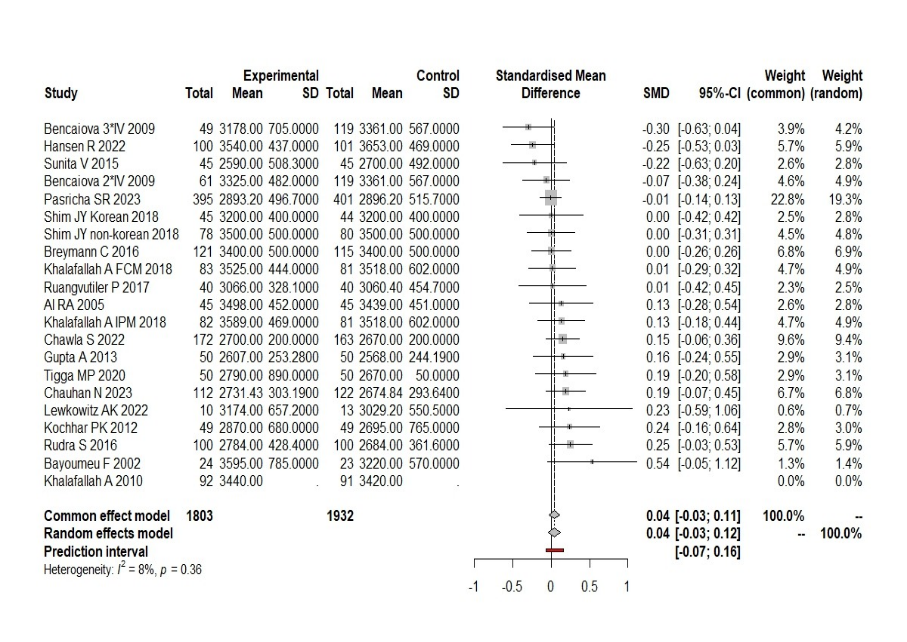
Eighteen studies reported the mean birth weight of the newborn. Bencaiova et al. (58), Shim J et.al(18), Khalafallah A et.al(19) and Shim JY had reported the findings from both the groups thus pooled analysis was performed for a total of 21 studies (n= 3,735) (Figure 5a) reporting birth weight as an outcome. Khalafallah A et.al 2010(20) did not report the standard deviation thus excluded from pooled results.


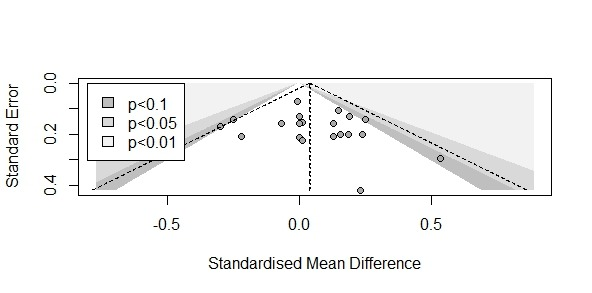


**aa**

**ba**

Figure 5 Meta-analysis of effect of IV iron versus oral iron. **a.** Forest plot showing the effect of IV versus oral iron on the birth weight of newborn; **b.** Funnel plot for estimates of publication bias.

Study by Abhilashini GD(14) and Chawla S(21) reported no significant differences in birth weight were found between the groups but no data were provided on request thus excluded. Utilizing random effect model, the pooled results showed no significant difference [SMD: 0.04 (95% CI: 0.03; 0.11)] in the birth weight in both the groups. We could not observe statistically significant heterogeneity among the studies (I^2^ = 8%; p=0.36) (Figure 5b).

Sensitivity analysis by removing studies with high risk of bias(5,21) and higher weight(10) revealed negligible difference in the pooled effect from the main model, indicating robustness of the main analysis. Symmetrical distribution of studies was observed through contour enhanced visual funnel plot, which was confirmed by Egger’s test depicting no publication bias (p = 0.4216).

1. **Cord Haemoglobin concentration:**


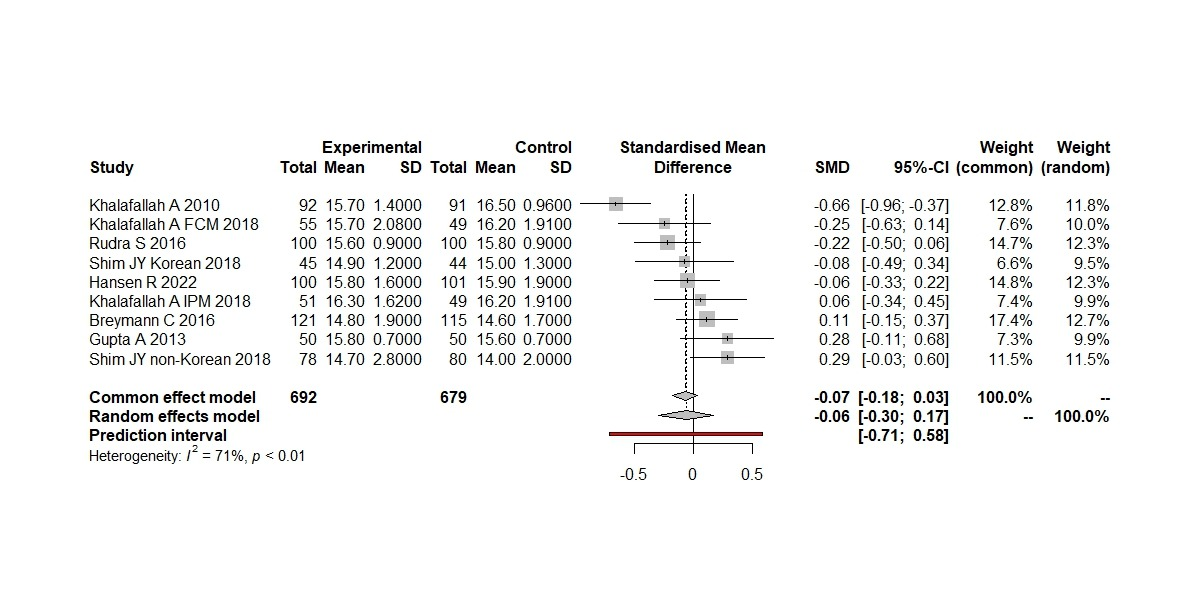
Utilizing the random effect model, pooled result from meta-analysis from 9 studies (n= 1371) suggests that there is no significant change in the cord haemoglobin [SMD: -0.06 (95% CI: -0.29; 0.17)] concentration amongst women in both the groups. We could observe statistically significant heterogeneity among the studies (I^2^ = 71%; p<0.01) (Figure 6a). When the studies with higher weight (11,16,22–24) were removed in sensitive analysis, non-significant reduction (21%) was observed in heterogeneity and pooled effect of standard mean difference became nil, indicating that the difference in the main model was only because of the studies with higher weight. Contour enhanced visual funnel plot showed symmetrical distribution of studies (Figure 6b).


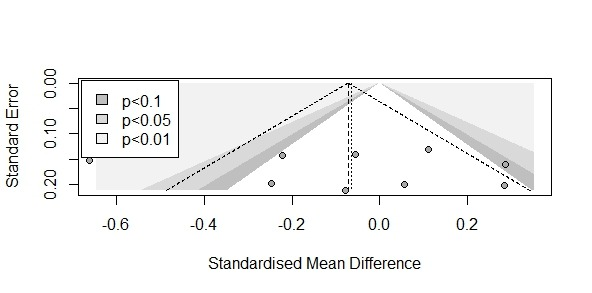


**ba**

**aa**

Figure 6 Meta-analysis of effect of IV iron versus oral iron. **a.** Forest plot showing the effect of IV versus oral iron on the cord haemoglobin concentration; **b**. Funnel plot for estimates in meta-analysis for publication bias.

1. **Length of newborn:**

Utilizing the random effect model**,** pooled findings from 6 studies (n= 1606) (findings from two common studies- Khalafallah et.al(20) and Shim JY(18) ) suggests that there is no significant difference in the length of newborn (SMD 0.02, 95% CI -0.01; 0.06) amongst women in both the groups. We could not observe significant heterogeneity among the studies (I^2^ = 0%; p=1.00) (Figure 7a). Sensitivity analysis by removing the higher weight studies(4,10,16) increased the pooled SMD to 0.05 (95% CI: -0.22; 0.32). We could not observe significant heterogeneity among the studies (I^2^ = 0%; p=1.00) (Figure 7b). Contour enhanced funnel plot showed asymmetrical distribution of studies. Imputation of two studies with trim and fill method showed negligible change in pooled SMD (0.013; 95% CI: 0.93;0.38).


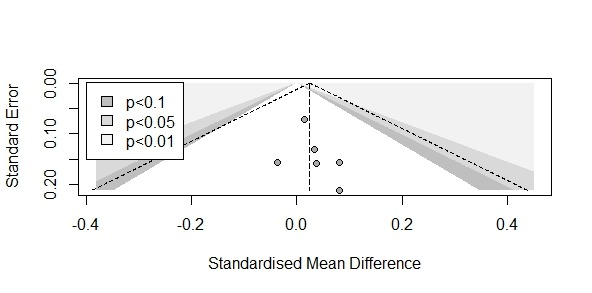

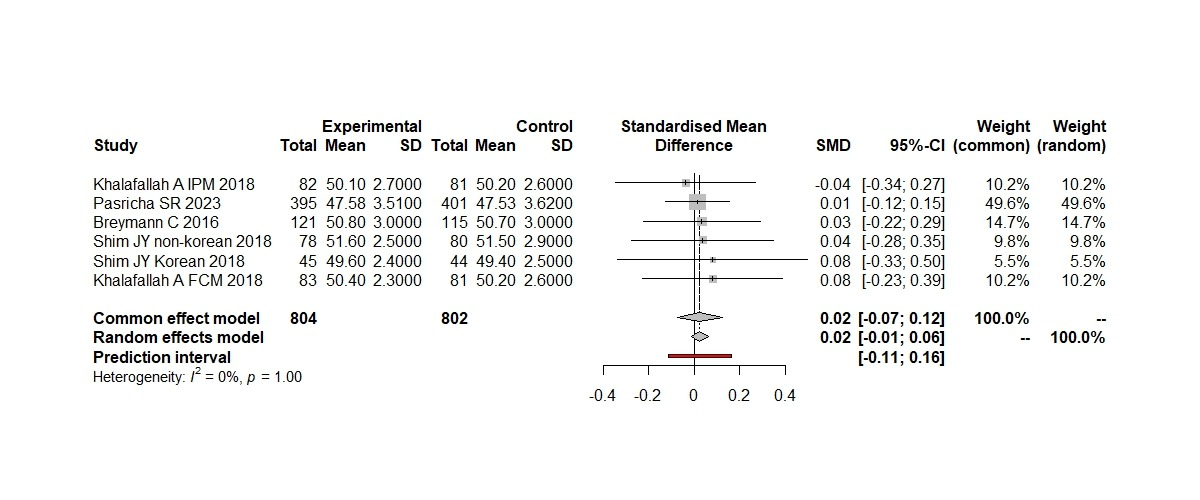


**ba**

**aa**

Figure 7 Meta-analysis of effect of IV iron versus oral iron. **a.** Forest plot showing the effect of IV versus oral iron on the length of newborn; **b.** Funnel plot for estimates in meta-analysis for publication bias.

1. **Still Birth and Neonatal Deaths:**

Of 34 selected studies, four studies (8,10,11,23) (n=3139 ) provided data on still births/intrauterine death and three studies (8,10,11) provided data on neonatal deaths. Utilizing random effect model, pooled meta-analysis showed no significant difference for still births [OR: 0.92 (95% CI: 0.55; 1.54)]. Similarly, for neonatal deaths (n=2844) no significant difference was observed (OR 0.72; 95% CI [0.44; 1.18]) (Figure 8a and 8c). In still birth, after excluding the studies with higher weight(10,23), no significant difference was observed in the pooled effect [OR 1.00 (95% CI: 0.10;9.69)] among both the groups indicating that no study have a considerable influence on the pooled estimate. In neonatal deaths, studies with higher weight influenced the pooled effect. Studies showed no significant heterogeneity for both the conditions (I^2^=0%; p = 0.79 and 0.68). However, the power of the heterogeneity test was very low, as there were only four and three studies in the respective outcomes. Contour enhanced funnel plot showed symmetrical distribution of studies for both still birth and neonatal deaths (Figure 8b and 8d).


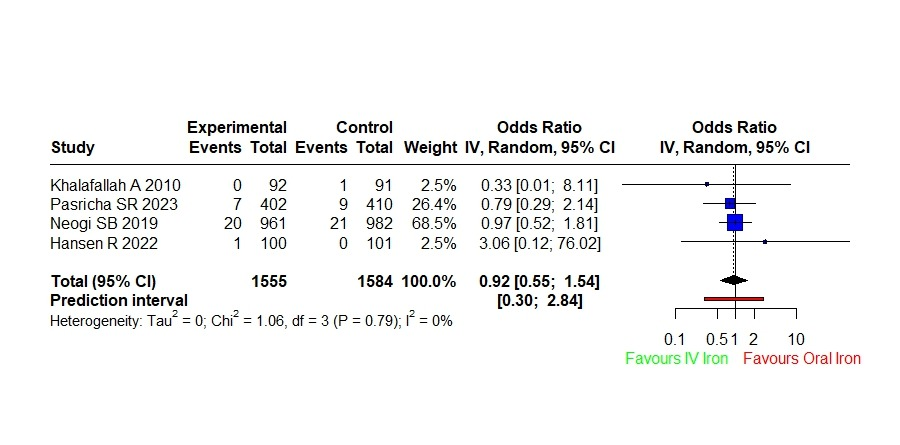


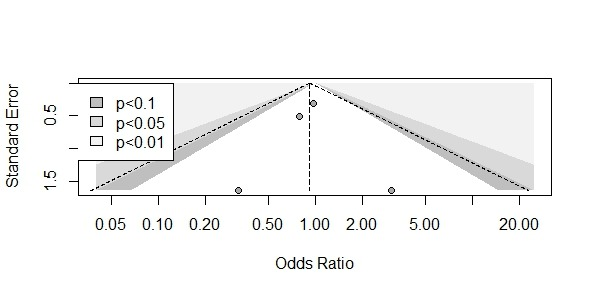


**aa**

**b**


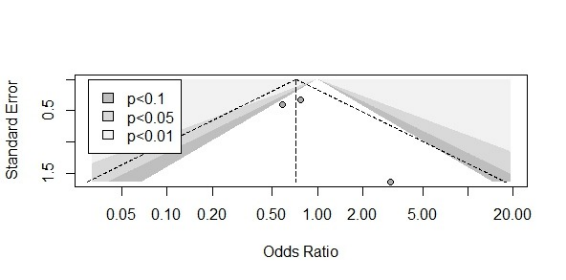

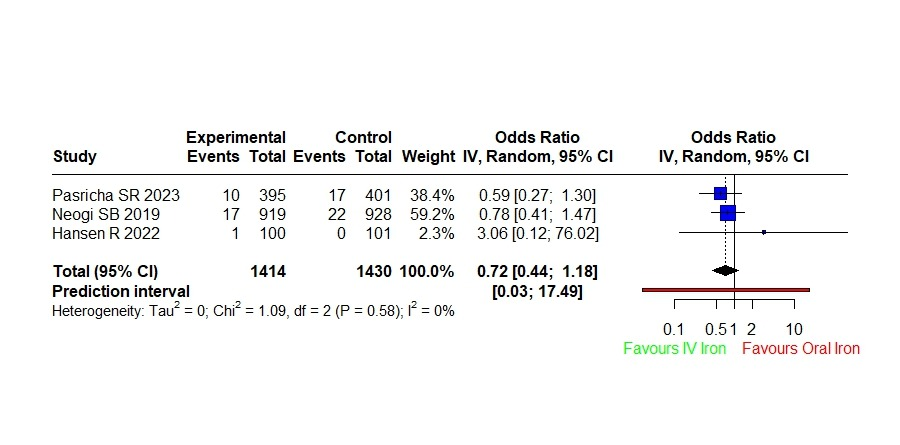


**ca**

**d**

Figure 8 Meta-analysis of effect of IV iron versus oral iron. **a.** Forest plot showing the effect of IV versus oral iron on still births; **b.** Funnel plot for estimates in meta-analysis for IV iron and still births versus oral iron and still births; c**.** Forest plot showing the effect of IV versus oral iron on neonatal deaths; **d.** Funnel plot for estimates in meta-analysis for IV iron and neonatal deaths versus oral iron and neonatal deaths.

1. **Pre-term births:**

Eight studies (4,5,8,10–12,16,25) provided data on pre-term birth in both the arms. Khalafallah A 2018 (4) reported the outcome for both of its IV groups (FCM and IPM), thus pooled analysis has been performed for a total of nine studies (n= 3738). Utilizing random effects model, pooled odds ratio showed almost negligible difference among both IV and oral iron arms (OR 0.97; 95% CI [0.79; 1.18]). No individual study showed statistically significant effect sizes for the outcome. No significant heterogeneity was observed among the studies (I^2^ = 0%; p=0.84) (Figure 9a). Sensitivity analysis by excluding high risk of bias studies revealed no difference in the pooled effect from the main model. However, removal of studies with higher weight considerably reduced the pooled OR to 0.62 (95% CI: 0.31; 1.24) but the effect was non-significant. No significant heterogeneity was observed among the studies (I^2^ = 0%; p=0.84) (Figure 9b). The contour enhanced funnel plot showed asymmetrical distribution of the studies, depicting presence of publication bias. Trim and fill effect imputed 5 missing studies. The adjusted result showed that the pooled effect of preterm birth changed from 0.97 to 0.99 (95% CI: 0.82; 1.21), which was not significant.


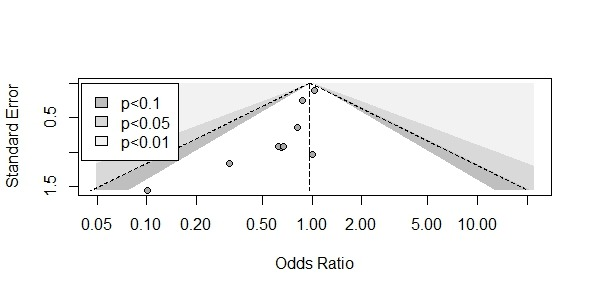

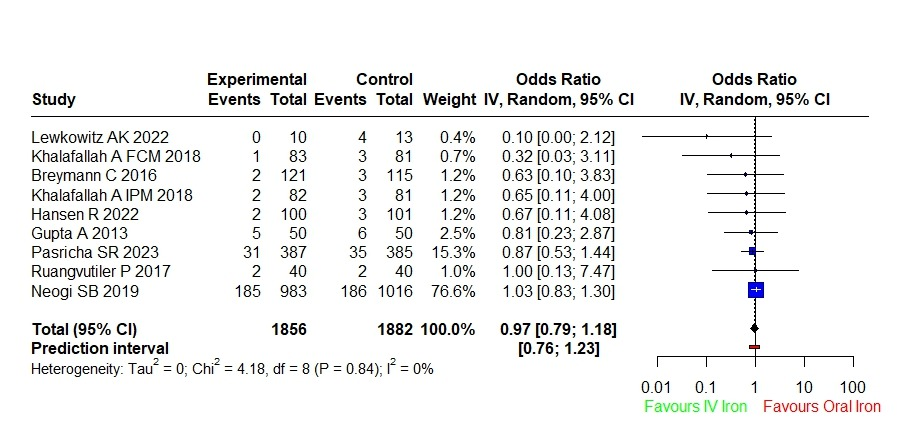


**aa**

**b**

Figure 9 Meta-analysis of effect of IV iron versus oral iron **a.** Forest plot showing the effect of IV versus oral iron on pre-term birth; **b.** Funnel plot for estimates in meta-analysis for IV iron and pre-term birth versus oral iron and pre-term birth.

**Reference:**

1. Al RA, Unlubilgin E, Kandemir O, Yalvac S, Cakir L, Haberal A. Intravenous versus oral iron for treatment of anemia in pregnancy: a randomized trial. Obstet Gynecol. 2005 Dec;106(6):1335–40.

2. Bayoumeu F, Subiran-Buisset C, Baka NE, Legagneur H, Monnier-Barbarino P, Laxenaire MC. Iron therapy in iron deficiency anemia in pregnancy: intravenous route versus oral route. Am J Obstet Gynecol. 2002 Mar;186(3):518–22.

3. Kochhar PK, Kaundal A, Ghosh P. Intravenous iron sucrose versus oral iron in treatment of iron deficiency anemia in pregnancy: A randomized clinical trial. Journal of Obstetrics and Gynaecology Research. 2013;39(2):504–10.

4. Khalafallah AA, Hyppa A, Chuang A, Hanna F, Wilson E, Kwok C, et al. A Prospective Randomised Controlled Trial of a Single Intravenous Infusion of Ferric Carboxymaltose vs Single Intravenous Iron Polymaltose or Daily Oral Ferrous Sulphate in the Treatment of Iron Deficiency Anaemia in Pregnancy. Semin Hematol. 2018 Oct;55(4):223–34.

5. Lewkowitz AK, Stout MJ, Cooke E, Deoni SC, D’Sa V, Rouse DJ, et al. Intravenous versus Oral Iron for Iron-Deficiency Anemia in Pregnancy (IVIDA): A Randomized Controlled Trial. Am J Perinatol. 2022 Jun;39(8):808–15.

6. Bencaiova G, von Mandach U, Zimmermann R. Iron prophylaxis in pregnancy: intravenous route versus oral route. Eur J Obstet Gynecol Reprod Biol. 2009 Jun;144(2):135–9.

7. Froessler B, Cocchiaro C, Saadat-Gilani K, Hodyl N, Dekker G. Intravenous iron sucrose versus oral iron ferrous sulfate for antenatal and postpartum iron deficiency anemia: a randomized trial. J Matern Fetal Neonatal Med. 2013 May;26(7):654–9.

8. Neogi SB, Devasenapathy N, Singh R, Bhushan H, Shah D, Divakar H, et al. Safety and effectiveness of intravenous iron sucrose versus standard oral iron therapy in pregnant women with moderate-to-severe anaemia in India: a multicentre, open-label, phase 3, randomised, controlled trial. The Lancet Global Health. 2019 Dec 1;7(12):e1706–16.

9. Sunita V, Kolekar R, Gundalli S, Nandurkar V. Effectiveness of Intravenous Iron Sucrose versus Oral Iron in Iron Deficiency Anemia in Pregnancy. IOSR Journal of Dental and Medical Sciences (IOSR-JDMS). 2015 Jan;14(1):52–60.

10. Pasricha SR, Mwangi MN, Moya E, Ataide R, Mzembe G, Harding R, et al. Ferric carboxymaltose versus standard-of-care oral iron to treat second-trimester anaemia in Malawian pregnant women: a randomised controlled trial. The Lancet. 2023 May 13;401(10388):1595–609.

11. Hansen R, Sommer VM, Pinborg A, Krebs L, Thomsen LL, Moos T, et al. Intravenous ferric derisomaltose versus oral iron for persistent iron deficient pregnant women: a randomised controlled trial. Arch Gynecol Obstet [Internet]. 2022 Sep 15 [cited 2023 Jul 26]; Available from: https://doi.org/10.1007/s00404-022-06768-x

12. Gupta A, Manaktala U, Rathore AM. A Randomised Controlled Trial to Compare Intravenous Iron Sucrose and Oral Iron in Treatment of Iron Deficiency Anemia in Pregnancy. Indian J Hematol Blood Transfus. 2014 Jun;30(2):120–5.

13. Singh K, Fong YF, Kuperan P. A comparison between intravenous iron polymaltose complex (Ferrum Hausmann®) and oral ferrous fumarate in the treatment of iron deficiency anaemia in pregnancy. European Journal of Haematology. 1998;60(2):119–24.

14. Abhilashini GD, Sagili H, Reddi R. Intravenous Iron Sucrose and Oral Iron for the Treatment of Iron Deficiency Anaemia in Pregnancy. J Clin Diagn Res. 2014 May;8(5):OC04–7.

15. Chauhan N, Dogra P, Sharma R, Kant S, Soni M. Randomized Controlled Trial Comparing Ferrous Sulfate and Iron Sucrose in Iron Deficiency Anemia in Pregnancy. Cureus. 15(2):e34858.

16. Breymann C, Milman N, Mezzacasa A, Bernard R, Dudenhausen J, FER-ASAP investigators. Ferric carboxymaltose vs. oral iron in the treatment of pregnant women with iron deficiency anemia: an international, open-label, randomized controlled trial (FER-ASAP). J Perinat Med. 2017 May 24;45(4):443–53.

17. Dubey S, Suri V, Aggarawal N, Das R. Is it safe to use intravenous iron sucrose during pregnancy? A randomized controlled trial. International Journal of Reproduction, Contraception, Obstetrics and Gynecology. 2013;2(4):544–9.

18. Shim JY, Kim MY, Kim YJ, Lee Y, Lee JJ, Jun JK, et al. Efficacy and safety of ferric carboxymaltose versus ferrous sulfate for iron deficiency anemia during pregnancy: subgroup analysis of Korean women. BMC Pregnancy Childbirth. 2018 Aug 28;18(1):349.

19. Khalafallah AA, Hyppa A, Chuang A, Hanna F, Wilson E, Kwok C, et al. A Prospective Randomised Controlled Trial of a Single Intravenous Infusion of Ferric Carboxymaltose vs Single Intravenous Iron Polymaltose or Daily Oral Ferrous Sulphate in the Treatment of Iron Deficiency Anaemia in Pregnancy. Semin Hematol. 2018 Oct;55(4):223–34.

20. Khalafallah A, Dennis A, Bates J, Bates G, Robertson IK, Smith L, et al. A prospective randomized, controlled trial of intravenous versus oral iron for moderate iron deficiency anaemia of pregnancy. J Intern Med. 2010 Sep;268(3):286–95.

21. Chawla S, Singh A, Jhamb D, Anupama CH. A Randomised Controlled Trial to Compare Injection Ferric Carboxymaltose and Oral Iron in Treating Iron Deficiency Anemia During Pregnancy. J Obstet Gynaecol India. 2022 Dec;72(6):492–6.

22. Rudra S, Chandna A, Nath J. Comparison of intravenous iron sucrose with oral iron in pregnant women with iron deficiency anaemia. International Journal of Reproduction, Contraception, Obstetrics and Gynecology. 2016;5(3):747–51.

23. Khalafallah A, Dennis A, Bates J, Bates G, Robertson IK, Smith L, et al. A prospective randomized, controlled trial of intravenous versus oral iron for moderate iron deficiency anaemia of pregnancy. J Intern Med. 2010 Sep;268(3):286–95.

24. Shim JY, Kim MY, Kim YJ, Lee Y, Lee JJ, Jun JK, et al. Efficacy and safety of ferric carboxymaltose versus ferrous sulfate for iron deficiency anemia during pregnancy: subgroup analysis of Korean women. BMC Pregnancy Childbirth. 2018 Aug 28;18(1):349.

25. Ruangvutilert P, Chanprapaph P, Chuenwattana P, Titapant V, Komoltri C. Low-Dose Weekly Intravenous Iron Sucrose versus Daily Oral Iron for Iron Deficiency Anemia in Late Pregnancy: A Randomized Controlled Trial. JOURNAL OF THE MEDICAL ASSOCIATION OF THAILAND. 2017 May 1;100(5):496.
