# Supplementary material for: Clinical outcome post treatment of anemia in pregnancy with intravenous versus oral iron therapy: a systematic review and meta-analysis
Source: Sci Rep. 2024 Jan 2;14:179. doi: 10.1038/s41598-023-50234-w (PMC10761955; doi:10.1038/s41598-023-50234-w)
Supplement: Supplementary file 7 — Supplementary Information 7. [file 41598_2023_50234_MOESM7_ESM.docx]

**Supplementary file 7**: GRADE summary of findings

| **Table 1:** Intravenous iron compared to Oral Iron for decreasing need for blood transfusion.  Bibliography: | | | | | | | | | | | |
| --- | --- | --- | --- | --- | --- | --- | --- | --- | --- | --- | --- |
| **Certainty assessment** | | | | | | | **Summary of findings** | | | | |
| **Participants (studies) Follow-up** | **Risk of bias** | **Inconsistency** | **Indirectness** | **Imprecision** | **Publication bias** | **Overall certainty of evidence** | **Study event rates (%)** | | **Relative effect (95% CI)** | **Anticipated absolute effects** | |
|  |  |  |  |  |  |  | **With Oral Iron** | **With Intravenous iron** |  | **Risk with Oral Iron** | **Risk difference with Intravenous iron** |
| **New outcome** | | | | | | | | | | | |
| 4194 (13 RCTs) | not serious | not serious | not serious | not serious | publication bias strongly suspected^a^ | ⨁⨁⨁◯ Moderate | 48/2167 (2.2%) | 35/2027 (1.7%) | **OR 0.81** (0.53 to 1.26) | 22 per 1,000 | **4 fewer per 1,000** (from 10 fewer to 6 more) |

**CI:** confidence interval; **OR:** odds ratio

#### Explanations

a. Studies showed asymmetry in its distribution through visual funnel plot, with more studies cluttered with less odds ratio and high standard error, thus reflecting the presence of publication bias, which was further confirmed by Egger’s test (p <0.001).

| **Table 2:** Intravenous iron compared to Oral Iron for reducing post-partum hemorrhage.  Bibliography: | | | | | | | | | | | |
| --- | --- | --- | --- | --- | --- | --- | --- | --- | --- | --- | --- |
| **Certainty assessment** | | | | | | | **Summary of findings** | | | | |
| **Participants (studies) Follow-up** | **Risk of bias** | **Inconsistency** | **Indirectness** | **Imprecision** | **Publication bias** | **Overall certainty of evidence** | **Study event rates (%)** | | **Relative effect (95% CI)** | **Anticipated absolute effects** | |
|  |  |  |  |  |  |  | **With Oral Iron** | **With Intravenous iron** |  | **Risk with Oral Iron** | **Risk difference with Intravenous iron** |
| **New outcome** | | | | | | | | | | | |
| 3655 (8 RCTs) | not serious | not serious | not serious | not serious | publication bias strongly suspected^a^ | ⨁⨁⨁◯ Moderate | 128/1909 (6.7%) | 84/1746 (4.8%) | **OR 1.01** (0.72 to 1.41) | 67 per 1,000 | **1 more per 1,000** (from 18 fewer to 25 more) |

**CI:** confidence interval; **OR:** odds ratio

#### Explanations

a. Studies showed asymmetry in its distribution through visual funnel plot.

| **Table 3:** Intravenous iron compared to Oral Iron for reducing caesarean section.  Bibliography: | | | | | | | | | | | |
| --- | --- | --- | --- | --- | --- | --- | --- | --- | --- | --- | --- |
| **Certainty assessment** | | | | | | | **Summary of findings** | | | | |
| **Participants (studies) Follow-up** | **Risk of bias** | **Inconsistency** | **Indirectness** | **Imprecision** | **Publication bias** | **Overall certainty of evidence** | **Study event rates (%)** | | **Relative effect (95% CI)** | **Anticipated absolute effects** | |
|  |  |  |  |  |  |  | **With Oral Iron** | **With Intravenous iron** |  | **Risk with Oral Iron** | **Risk difference with Intravenous iron** |
| **New outcome** | | | | | | | | | | | |
| 1813 (9 RCTs) | not serious | not serious | not serious | not serious | publication bias strongly suspected^a^ | ⨁⨁⨁◯ Moderate | 187/972 (19.2%) | 153/841 (18.2%) | **OR 1.10** (0.86 to 1.42) | 192 per 1,000 | **15 more per 1,000** (from 22 fewer to 60 more) |

**CI:** confidence interval; **OR:** odds ratio

#### Explanations

a. The visual funnel plot showed asymmetrical distribution of the studies for CS delivery.

| **Table 4:** Intravenous iron compared to Oral Iron for reducing assisted/instrumental delivery.  Bibliography: | | | | | | | | | | | |
| --- | --- | --- | --- | --- | --- | --- | --- | --- | --- | --- | --- |
| **Certainty assessment** | | | | | | | **Summary of findings** | | | | |
| **Participants (studies) Follow-up** | **Risk of bias** | **Inconsistency** | **Indirectness** | **Imprecision** | **Publication bias** | **Overall certainty of evidence** | **Study event rates (%)** | | **Relative effect (95% CI)** | **Anticipated absolute effects** | |
|  |  |  |  |  |  |  | **With Oral Iron** | **With Intravenous iron** |  | **Risk with Oral Iron** | **Risk difference with Intravenous iron** |
| **New outcome** | | | | | | | | | | | |
| 1019 (5 RCTs) | not serious | not serious | not serious | not serious | publication bias strongly suspected^a^ | ⨁⨁⨁◯ Moderate | 31/576 (5.4%) | 27/443 (6.1%) | **OR 1.27** (0.70 to 2.27) | 54 per 1,000 | **14 more per 1,000** (from 16 fewer to 61 more) |

**CI:** confidence interval; **OR:** odds ratio

#### Explanations

a. The visual funnel plot showed asymmetrical distribution of the studies for assisted/instrumental delivery.

| **Table 5:** Intravenous iron compared to Oral Iron for reducing hypertensive disorders.  Bibliography: | | | | | | | | | | | |
| --- | --- | --- | --- | --- | --- | --- | --- | --- | --- | --- | --- |
| **Certainty assessment** | | | | | | | **Summary of findings** | | | | |
| **Participants (studies) Follow-up** | **Risk of bias** | **Inconsistency** | **Indirectness** | **Imprecision** | **Publication bias** | **Overall certainty of evidence** | **Study event rates (%)** | | **Relative effect (95% CI)** | **Anticipated absolute effects** | |
|  |  |  |  |  |  |  | **With Oral Iron** | **With Intravenous iron** |  | **Risk with Oral Iron** | **Risk difference with Intravenous iron** |
| **New outcome** | | | | | | | | | | | |
| 987 (5 RCTs) | not serious | not serious | not serious | not serious | publication bias strongly suspected^a^ | ⨁⨁⨁◯ Moderate | 19/491 (3.9%) | 9/496 (1.8%) | **OR 0.46** (0.20 to 1.08) | 39 per 1,000 | **21 fewer per 1,000** (from 31 fewer to 3 more) |

**CI:** confidence interval; **OR:** odds ratio

#### Explanations

a. The visual funnel plot shows the asymmetrical distribution of the studies.

| **Table 6:** Intravenous iron compared to Oral Iron for adequate birth weight.  Bibliography: | | | | | | | | | | | |
| --- | --- | --- | --- | --- | --- | --- | --- | --- | --- | --- | --- |
| **Certainty assessment** | | | | | | | **Summary of findings** | | | | |
| **Participants (studies) Follow-up** | **Risk of bias** | **Inconsistency** | **Indirectness** | **Imprecision** | **Publication bias** | **Overall certainty of evidence** | **Study event rates (%)** | | **Relative effect (95% CI)** | **Anticipated absolute effects** | |
|  |  |  |  |  |  |  | **With Oral Iron** | **With Intravenous iron** |  | **Risk with Oral Iron** | **Risk difference with Intravenous iron** |
| **New outcome** | | | | | | | | | | | |
| 3735 (21 RCTs) | not serious | not serious | not serious | not serious | none | ⨁⨁⨁⨁ High | -/1932 | -/1803 | not estimable | 0 per 1,000 |  |

**CI:** confidence interval

| **Table 7:** Intravenous iron compared to Oral Iron for normal cord Haemoglobin concentration.  Bibliography: | | | | | | | | | | | |
| --- | --- | --- | --- | --- | --- | --- | --- | --- | --- | --- | --- |
| **Certainty assessment** | | | | | | | **Summary of findings** | | | | |
| **Participants (studies) Follow-up** | **Risk of bias** | **Inconsistency** | **Indirectness** | **Imprecision** | **Publication bias** | **Overall certainty of evidence** | **Study event rates (%)** | | **Relative effect (95% CI)** | **Anticipated absolute effects** | |
|  |  |  |  |  |  |  | **With Oral Iron** | **With Intravenous iron** |  | **Risk with Oral Iron** | **Risk difference with Intravenous iron** |
| **New outcome** | | | | | | | | | | | |
| 1371 (9 RCTs) | not serious | not serious | not serious | not serious | none | ⨁⨁⨁⨁ High | -/679 | -/692 | not estimable | 0 per 1,000 |  |

**CI:** confidence interval

| **Table 8:** Intravenous iron compared to Oral Iron for reducing preterm birth.  Bibliography: | | | | | | | | | | | |
| --- | --- | --- | --- | --- | --- | --- | --- | --- | --- | --- | --- |
| **Certainty assessment** | | | | | | | **Summary of findings** | | | | |
| **Participants (studies) Follow-up** | **Risk of bias** | **Inconsistency** | **Indirectness** | **Imprecision** | **Publication bias** | **Overall certainty of evidence** | **Study event rates (%)** | | **Relative effect (95% CI)** | **Anticipated absolute effects** | |
|  |  |  |  |  |  |  | **With Oral Iron** | **With Intravenous iron** |  | **Risk with Oral Iron** | **Risk difference with Intravenous iron** |
| **New outcome** | | | | | | | | | | | |
| 3738 (9 RCTs) | not serious | not serious | not serious | not serious | publication bias strongly suspected^a^ | ⨁⨁⨁◯ Moderate | 245/1882 (13.0%) | 230/1856 (12.4%) | **OR 0.97** (0.79 to 1.18) | 130 per 1,000 | **3 fewer per 1,000** (from 24 fewer to 20 more) |

**CI:** confidence interval; **OR:** odds ratio

#### Explanations

a. Visual funnel plot showed asymmetrical distribution of studies thus depicting publication bias.

| **Table 9:** Intravenous iron compared to Oral Iron for normal length of newborn.  Bibliography: | | | | | | | | | | | |
| --- | --- | --- | --- | --- | --- | --- | --- | --- | --- | --- | --- |
| **Certainty assessment** | | | | | | | **Summary of findings** | | | | |
| **Participants (studies) Follow-up** | **Risk of bias** | **Inconsistency** | **Indirectness** | **Imprecision** | **Publication bias** | **Overall certainty of evidence** | **Study event rates (%)** | | **Relative effect (95% CI)** | **Anticipated absolute effects** | |
|  |  |  |  |  |  |  | **With Oral Iron** | **With Intravenous iron** |  | **Risk with Oral Iron** | **Risk difference with Intravenous iron** |
| **New outcome** | | | | | | | | | | | |
| 1606 (6 RCTs) | not serious | not serious | not serious | not serious | publication bias strongly suspected^a^ | ⨁⨁⨁◯ Moderate | -/802 | -/804 | not estimable | 0 per 1,000 |  |

**CI:** confidence interval

#### Explanations

a. Visual funnel plot showed asymmetrical distribution of the studies.

| **Table 10:** Intravenous iron compared to Oral Iron for reducing stillbirth.  Bibliography: | | | | | | | | | | | |
| --- | --- | --- | --- | --- | --- | --- | --- | --- | --- | --- | --- |
| **Certainty assessment** | | | | | | | **Summary of findings** | | | | |
| **Participants (studies) Follow-up** | **Risk of bias** | **Inconsistency** | **Indirectness** | **Imprecision** | **Publication bias** | **Overall certainty of evidence** | **Study event rates (%)** | | **Relative effect (95% CI)** | **Anticipated absolute effects** | |
|  |  |  |  |  |  |  | **With Oral Iron** | **With Intravenous iron** |  | **Risk with Oral Iron** | **Risk difference with Intravenous iron** |
| **New outcome** | | | | | | | | | | | |
| 3139 (4 RCTs) | not serious | not serious | not serious | not serious | none | ⨁⨁⨁⨁ High | 31/1584 (2.0%) | 28/1555 (1.8%) | **OR 0.92** (0.55 to 1.54) | 20 per 1,000 | **2 fewer per 1,000** (from 9 fewer to 10 more) |

**CI:** confidence interval; **OR:** odds ratio

| **Table 11:** Intravenous iron compared to Oral Iron for reducing neonatal death.  Bibliography: | | | | | | | | | | | |
| --- | --- | --- | --- | --- | --- | --- | --- | --- | --- | --- | --- |
| **Certainty assessment** | | | | | | | **Summary of findings** | | | | |
| **Participants (studies) Follow-up** | **Risk of bias** | **Inconsistency** | **Indirectness** | **Imprecision** | **Publication bias** | **Overall certainty of evidence** | **Study event rates (%)** | | **Relative effect (95% CI)** | **Anticipated absolute effects** | |
|  |  |  |  |  |  |  | **With Oral Iron** | **With Intravenous iron** |  | **Risk with Oral Iron** | **Risk difference with Intravenous iron** |
| **New outcome** | | | | | | | | | | | |
| 2844 (3 RCTs) | not serious | not serious | not serious | not serious | none | ⨁⨁⨁⨁ High | 39/1430 (2.7%) | 28/1414 (2.0%) | **OR 0.72** (0.44 to 1.18) | 27 per 1,000 | **7 fewer per 1,000** (from 15 fewer to 5 more) |

**CI:** confidence interval; **OR:** odds ratio
